# Organ-, sex- and age-dependent patterns of endogenous L1 mRNA expression at a single locus resolution

**DOI:** 10.1093/nar/gkab369

**Published:** 2021-05-22

**Authors:** Emily C Stow, Tiffany Kaul, Dawn L deHaro, Madeleine R Dem, Anna G Beletsky, Maria E Morales, Qianhui Du, Alexis J LaRosa, Hanlin Yang, Emily Smither, Melody Baddoo, Nathan Ungerleider, Prescott Deininger, Victoria P Belancio

**Affiliations:** Tulane Cancer Center, Tulane Health Sciences Center, 1700 Tulane Ave, New Orleans, LA 70112, USA; Department of Structural and Cellular Biology, Tulane School of Medicine, 1430 Tulane Ave, New Orleans, LA 70112 USA; Tulane Cancer Center, Tulane Health Sciences Center, 1700 Tulane Ave, New Orleans, LA 70112, USA; Department of Epidemiology, Tulane School of Public Health and Tropical Medicine, New Orleans, LA 70112 USA; Tulane Cancer Center, Tulane Health Sciences Center, 1700 Tulane Ave, New Orleans, LA 70112, USA; Department of Structural and Cellular Biology, Tulane School of Medicine, 1430 Tulane Ave, New Orleans, LA 70112 USA; Tulane Cancer Center, Tulane Health Sciences Center, 1700 Tulane Ave, New Orleans, LA 70112, USA; Department of Structural and Cellular Biology, Tulane School of Medicine, 1430 Tulane Ave, New Orleans, LA 70112 USA; Tulane Cancer Center, Tulane Health Sciences Center, 1700 Tulane Ave, New Orleans, LA 70112, USA; Department of Structural and Cellular Biology, Tulane School of Medicine, 1430 Tulane Ave, New Orleans, LA 70112 USA; Tulane Cancer Center, Tulane Health Sciences Center, 1700 Tulane Ave, New Orleans, LA 70112, USA; Department of Epidemiology, Tulane School of Public Health and Tropical Medicine, New Orleans, LA 70112 USA; Tulane Cancer Center, Tulane Health Sciences Center, 1700 Tulane Ave, New Orleans, LA 70112, USA; Department of Structural and Cellular Biology, Tulane School of Medicine, 1430 Tulane Ave, New Orleans, LA 70112 USA; Department of Structural and Cellular Biology, Tulane School of Medicine, 1430 Tulane Ave, New Orleans, LA 70112 USA; Tulane Cancer Center, Tulane Health Sciences Center, 1700 Tulane Ave, New Orleans, LA 70112, USA; Department of Structural and Cellular Biology, Tulane School of Medicine, 1430 Tulane Ave, New Orleans, LA 70112 USA; Tulane Cancer Center, Tulane Health Sciences Center, 1700 Tulane Ave, New Orleans, LA 70112, USA; Tulane Cancer Center, Tulane Health Sciences Center, 1700 Tulane Ave, New Orleans, LA 70112, USA; Tulane Cancer Center, Tulane Health Sciences Center, 1700 Tulane Ave, New Orleans, LA 70112, USA; Department of Epidemiology, Tulane School of Public Health and Tropical Medicine, New Orleans, LA 70112 USA; Tulane Cancer Center, Tulane Health Sciences Center, 1700 Tulane Ave, New Orleans, LA 70112, USA; Department of Structural and Cellular Biology, Tulane School of Medicine, 1430 Tulane Ave, New Orleans, LA 70112 USA

## Abstract

Expression of L1 mRNA, the first step in the L1 copy-and-paste amplification cycle, is a prerequisite for L1-associated genomic instability. We used a reported stringent bioinformatics method to parse L1 mRNA transcripts and measure the level of L1 mRNA expressed in mouse and rat organs at a locus-specific resolution. This analysis determined that mRNA expression of L1 loci in rodents exhibits striking organ specificity with less than 0.8% of loci shared between organs of the same organism. This organ specificity in L1 mRNA expression is preserved in male and female mice and across age groups. We discovered notable differences in L1 mRNA expression between sexes with only 5% of expressed L1 loci shared between male and female mice. Moreover, we report that the levels of total L1 mRNA expression and the number and spectrum of expressed L1 loci fluctuate with age as independent variables, demonstrating different patterns in different organs and sexes. Overall, our comparisons between organs and sexes and across ages ranging from 2 to 22 months establish previously unforeseen dynamic changes in L1 mRNA expression *in vivo*. These findings establish the beginning of an atlas of endogenous L1 mRNA expression across a broad range of biological variables that will guide future studies.

## INTRODUCTION

Long interspersed element-1 (LINE-1 or L1) expression can result in DNA damage and insertions, which may lead to various biological consequences such as mutagenesis, apoptosis, senescence, or changes in gene expression (1–12). L1 DNA makes up ∼17% of the human genome with over 500 000 copies and ∼18% of the mouse genome with ∼600 000 L1 fragments (13–15). Of the 500 000 human L1 copies, ∼5000 have an intact promoter within the 5′ UTR and only 80–120 are capable of retrotransposition in a given human (1,15–17). Unlike the human L1s, retrotransposition of which has declined over the last 40 Myr, mouse L1 elements have retained a more constant rate of retrotransposition over time (13–15). Functional studies have revealed that about 3000 mouse L1s remain retrotransposition-competent (14). Full-length, retrotransposition-competent L1 elements are about 6 kb in humans and 7 kb in mice, contain 5′ and 3′ untranslated regions, and two open reading frames (ORF1 and ORF2) (12,18–22). ORF1p contains RNA chaperoning activity and typically associates with L1-specific RNA molecules (20,23). ORF2p contains the endonuclease and reverse transcriptase enzymatic domains both of which are required for retrotransposition (21–24). The primary structural difference between mouse and human L1 elements is that the 5′ UTR of the mouse element varies in length and is made up of repetitive monomers (12,19,25,26). Mouse L1 elements are classified into subfamilies that are distinguished by the sequence of their monomeric 5′ repeats but are otherwise 97% identical (25,26). Three L1 subfamilies in mouse genomes are currently active: A, T_f_, and G_f_ (13,26–29). Like the mouse L1, the rat L1 has a 5′ UTR promoter region that consists of tandem arrays of monomers (30–32). L1 sequences make up 23% of the rat genome (32). Even though mice and rats are widely used model organisms for medical and evolutionary studies, there is sparse to no information about the endogenous mRNA expression of L1 elements at the locus-specific level in these or other organisms. Recently, techniques have been developed to analyze L1 mRNA expression and they reveal that different, small subsets of L1s are expressed in different human cell lines (33–36). Here, we implement our techniques to study patterns of L1 mRNA expression in mice and rats at a locus-specific level to determine patterns of L1 expression from normal organs, *in vivo*.

L1 sequences can be found in cellular RNA pools in two major forms. One form, which we will refer to as L1 mRNA, is transcribed from the 5′ UTR promoter of a full-length L1 locus (34,37,38). These L1 mRNA transcripts are a required part of the retrotransposition life cycle and give rise to *de novo* L1 inserts (6,18,23,39). Human, mouse, and rat L1 elements undergo the same steps in their replication cycle leading to *de novo* integration as the final outcome of their expression (1,14,40). The other L1-related RNA species come from full-length or truncated L1 sequences incorporated into cellular mRNA during RNA Pol II transcription of cellular genes. These L1 sequences are primarily L1 fragments embedded in the introns and 3′ UTRs of genes (13,15,32). These passively transcribed L1 sequences are as much as 100-fold more abundant than L1 mRNA, which creates a technical barrier in detecting L1 mRNA expression (33,34,38,41,42). Although L1 sequences incorporated into cellular transcripts can have significant biological implications on cell function by creating novel hybrid transcripts through alternative splicing and polyadenylation (6–10,12,18,43), they are still considered background sequences for the purposes of understanding the retrotransposition process. Thus, L1 mRNA expression from its own promoter is an important first step in the L1 amplification cycle.

Understanding patterns of endogenous L1 mRNA expression *in vivo* is important for determining where and when an insult to the host genome from these elements might occur. Ongoing L1 expression is expected to increase the likelihood of insertions, some of which have negative implications in mammalian health (1,39,44). Human retroelement insertions give rise to 0.3% of all new germ line human genetic diseases (45–47). Approximately 1% of colorectal cancer patients have a *de novo* somatic integration in the APC gene, which is believed to be a cancer-initiating event in a normal somatic cell (48,49). Independent of or through the process of retrotransposition, L1s can create DNA double-strand breaks (2,3) which may play a role in non-allelic homologous recombination (17,50,51) and lead to apoptosis (3,5,52) or senescence (4,5). In addition to increased L1 expression and/or retrotransposition in many human cancers (39,42,53–55), L1 expression and the resulting impact on host cells *in vivo* could be influenced by environmental exposures and physiologic stresses (35,36,41,52,56–60). Despite significant variation in the number of L1 retrotransposition events in the same tumor types developed in different individuals (55,61–63), it is not known whether interpersonal or sex-specific changes in L1 expression exist or potentially contribute to these observed differences. Similar to humans, mouse genomes experience mutations associated with L1 retrotransposition. Active L1 retrotransposition in mice has produced six stable mutants via *de novo* insertion events (14,28,64,65). Also, 6723 putative L1 polymorphisms have been detected in the C57BL/6 laboratory mouse strain (66). As ongoing L1 retrotransposition requires L1 mRNA expression, laboratory mice make an appropriate model to investigate patterns of L1 mRNA expression in different organs, sexes, and chronologies.

Despite evidence of significant health impacts of expressed L1 elements, little is understood about endogenous L1 transcription across the genome at the locus-specific level. Understanding locus-specific expression of L1 is critical because each locus is almost certainly dependent on its unique genomic environment. Furthermore, sequence variation between different loci makes the relative potential for retrotransposition vary considerably between loci. Thus, the insertion rate from a locus is proportional to both its ability to make L1 mRNA combined with its sequence-specific ‘hotness’ (1,16,39). By adapting our locus-specific L1 mRNA expression approach (34–36) to the mouse and rat genomes to rigorously eliminate passive transcription, we are able to see remarkable tissue-specificity in terms of expressed loci, that is also influenced by sex and age of the animals.

## MATERIALS AND METHODS

### Mouse and rat samples

The mice used in this study are C57BL/6. The rats used in this study are strain F344. Testes, brains, and livers were collected from 6 mice: 3 at 7.6 months old (mo) and 3 at 12.8 mo. Lungs were collected from 5 mice: 2 at 7.6 mo and 3 at 12.8 mo. Only testes and livers were collected from 6 additional mice: 2 at 1.9 mo, 1 at 3.6 mo, and 3 at 22.3 mo. Brains, lungs and ovaries were collected from nine mice: two at 2.2 mo, one at 3.2 mo, two at 8 mo, one at 8.6 mo, one at 8.7 mo and two at 16.5 mo. Uteri were collected from six mice: one at 2.2 mo, one at 8 mo, one at 8.6 mo, one at 8.7 mo and two at 16.5 mo. Testes, brains, livers, and lungs were collected from three rats, all 4.8 mo. Animals used in this study and their inclusion in downstream analyses is shown in Supplementary Figure S3.

### Cytoplasmic RNA preparation

RNA was extracted as previously described (34–36). Specifically, organ samples of ∼10 mm^3^ were bluntly dissected and then homogenized in 500–1000 ul of lysis buffer (150 mM NaCl, 50 mM HEPES pH 7.4, 25 ug/ml digitonin with 1000 U/ml SUPERase-In RNase inhibitor added just prior to application) to lyse the cytoplasmic membrane. The mixture was lightly homogenized in a Dounce homogenizer with an autoclaved B pestle, incubated on ice for 5 min and then centrifuged for 2 min at 1000 rpm at 4°C. Supernatant, containing the cytoplasmic fraction, was mixed with pre-chilled 7.5 ml of Trizol and 1.5 ml of chloroform and then centrifuged for 35 min at 4000 rpm at 4°C. The aqueous portion was transferred to 4.5 ml of chilled chloroform, mixed and centrifuged for 10 min at 4000 rpm at 4°C. The resulting aqueous portion was precipitated with 4.5 ml of isopropanol overnight at 80°C, centrifuged for 45 min at 4°C at 4000 rpm, washed with 10 ml of ethanol and re-suspended in RNase-free water.

### RNA quality check

Cytoplasmic RNA samples were analyzed in an Agilent 2100 Bioanalyzer System according to the Agilent RNA 6000 Nano kit guide. Samples with RIN >8 were submitted for sequencing.

### RNA sequencing

Cytoplasmic RNA samples were submitted to BGI Genomics for selection of polyadenylated RNAs, and strand-specific, paired-end library preparation. During this study, we used two paired-end RNA sequencing methods with the same read depth: DNA nanoball sequencing (DNBseq), which produces 150 bp read lengths, and Illumina sequencing, which produces 100 bp read lengths. We clipped 150 bp DNBseq sample reads to 100 bp and completed the analysis for the 150 bp reads and 100 bp reads in parallel to test whether these read length differences would affect the results. The analysis with different read lengths resulted in a difference of two authentically expressed L1 loci between the groups with 150bp reads producing 14 expressed L1 loci and 100bp reads producing 12 expressed L1 loci. For samples sequenced using Illumina sequencing, samples were pooled in groups of 5–7 and applied to a single lane of an Illumina HiSeq 2500/4000 instrument. Data were sorted based on barcodes attached to each individual sample, aligned to either the *Mus musculus* 10 (mm10) or *Rattus norvegicus* 6 (rn6) genomes, and queried for alignments occurring within annotated L1 loci. Bioinformatics analysis was performed as previously described (34–36). These methods are outlined in more detail below.

### Mus musculus annotations for full-length L1s

The annotation for full-length L1 elements was downloaded from the L1Base 2 database (67), including the annotations for both intact and non-intact (mutated open reading frames) elements. The full list of mm10 L1 coordinates is available in Supplemental File 1. Mouse L1 subfamilies and the number of L1 promoter monomers were also derived from L1Base 2 (67).

### Rattus norvegicus annotation for full-length L1s

The annotation for full-length, intact and non-intact L1 elements was downloaded from the L1Base 2 database (67) and intersected using BEDTools v2.27.1 (68) with coordinates of the BLAST search of the monomer repeat associated with the active L1 subfamily, L1Rn (31,69). The full list of rn6 L1 coordinates is available in Supplemental File 2.

### Bioinformatics analysis

The alignment strategy for RNA-Seq data to the genome of interest for identification of endogenous L1 mRNA expression studies has been previously described (34). Briefly, in this study we used Bowtie v0.12.8 (70) to concordantly map read pairs (-X 600) with a single preferred mapping location (-m 1) with the tryhard switch (-y) that forces it to exhaustively search each read pair against the reference genome, and allow up to three mismatches per alignment (-v 3). The same alignment parameters were used in this study with either mm10 or rn6 genomes. The generated bam file was strand separated and then intersected using BEDTools v2.27.1 with the same-oriented annotation for full-length L1s (68). Supplemental File 3 contains scripts for alignment of fastq files and extraction of reads corresponding to L1 loci coordinates. Manual curation of resulting L1 loci with greater than 10 mapped reads was performed using IGV and previously described criteria (36). Reads discarded by the unique (-m 1) and tryhard (-y) alignment parameters were collected (-un). The discarded reads were then aligned to T_f_, G_f_, and A subfamily sequences, each containing two subfamily-specific promoter monomers. The paired-end alignment was performed using STAR v2.3.0e, allowing two alignments and 25 mismatches per read pair. Reads that aligned to the promoter regions in the sense orientation were included in our analysis of discarded reads. RSEM v1.2.31 was used to measure the expression of genes containing L1 loci and total RNA levels (71). EBSeq v1.2.0 was used for differential expression analysis between uterus samples (72).

### Assessment of spliced transcripts

To assess whether transcription through an L1 locus was potentially due to spurious splicing with non-L1 transcripts during manual curation, the RNA-Seq samples were also aligned using the STAR program with default parameters for paired-end sequence files (73). The total number of splice events per sample was derived from the Log.final STAR output file. The number of splice junctions per expressed L1 locus was derived from the SJ.out.tab STAR output file.

### Normalization of transcript reads

To compare expression at the locus-specific level among multiple sequenced samples, the raw transcript reads mapping to each manually curated L1 loci were then normalized by calculating individual L1 loci fragments per thousand bases per million reads (FPKM) (36). As the L1 is ∼6 kb in length, the FPKM value was calculated by dividing the number of uniquely mapped transcript reads to an individual L1 locus and the product of the million mapped reads specific to the sequence sample of interest and 6. The described formula is shown here:

$$\begin{eqnarray*}&& {\rm FPKM}\;{\rm of}\;{\rm L1}\;{\rm locus}\;z = \nonumber \\ && \frac{{\# \;{\rm of}\;{\rm uniquely}\;{\rm mapped}\;{\rm reads}\;{\rm to}\;{\rm L1}\;{\rm locus}\;z\;{\rm in}\;{\rm sample}\;y}}{{{\rm million}\;{\rm mapped}\;{\rm reads}\;{\rm in}\;{\rm sample}\;y\; \times \;6}} \end{eqnarray*}$$

### Mappability assessment

To better understand and assess how ‘mappable’ L1 regions are in the mm10 and rn6 genomes, we downloaded species-specific whole genome Illumina paired-end sequencing files from NCBI. The sequencing files used are listed under NCBI SRA accession numbers SRR1382188 for the *Mus musculus* whole genome sequencing and ERR224469 for the *Rattus norvegicus* whole genome sequencing. Then we used the Bowtie1 alignment program to assign whole genome reads that mapped uniquely to the *Mus musculus* 10 (mm10) or *Rattus norvegicus* 6 (rn6) genomes (70), applying the same mapping strategy as for the RNA-Seq data, described above.

### Transcriptomic profiling

RNA-sequencing fastq files were processed using Kallisto v0.46.0 (74). Sleuth v0.30.0 (Wald test) was used to calculate differential expression values including fold change (FC) and false discovery rate (FDR) (75). These data were then used for gene set enrichment analysis (GSEA).

### Calculating mappability correction

To correct for potential underestimation in expression quantity of L1 loci due to the bowtie alignment parameter m -1, the uniquely mapped reads of the expressed L1 loci identified after manual curation were scaled to reflect the overall mappability of each locus. This correction factor was created by first using BEDTools v2.27.1 to extract the number of uniquely mapped reads from the mouse genomic .bam file that aligned to all full-length L1 loci and then graph those loci from highest to lowest mapped transcript reads (Supplementary Figure S11A) (68). As observed in Supplementary Figure S11A, on average, L1s with 400 reads had full coverage mappability. The number of reads able to map to a L1 locus in mouse genomic sequencing sample was scaled relative to 400 reads and that scaled number was then multiplied to the number of reads that mapped to each authentically expressed L1 locus in the RNA-Seq mouse samples. The described formula is shown here:

$$\begin{eqnarray*}&& {\rm Mappability}\;{\rm corrected}\;{\rm FPKM} = \nonumber \\ && {\rm FPKM}\; \times \;\left( {\frac{{400}}{{\# \;{\rm mapped}\;{\rm reads}\;{\rm from}\;{\rm DNA}{\hbox{-}}{\rm seq}}}} \right) \end{eqnarray*}$$

### Statistical analysis

Data are presented as mean with standard error bars. Data were analyzed by two-tailed Student's *t*-test when making comparisons between two groups. Chi-square tests were used when comparing categorical groups. To measure linear correlation between two variables, Pearson correlation test was performed. Statistical analysis was performed with GraphPad Prism.

## RESULTS

### Alignment and manual validation of L1 mRNA expression

We previously reported a stringent, custom-designed strategy for aligning and validating L1 mRNA reads originating from full-length L1 loci (34–36). For this approach, we harvested cytoplasmic RNA, selected polyadenylated transcripts, and performed strand-specific paired-end Next-Generation sequencing. Resulting RNA-Seq reads were analyzed using our published strategy (36) which includes stringent alignment parameters in the Bowtie1 aligner program (Supplementary Figure S1, see Materials and Methods for details). Due to the highly repetitive nature of L1 sequences, we utilized alignment settings to ensure that only unique and high-confidence alignments were reported. This approach eliminates a significant amount of the background caused by the multi-mapping of reads originating from both truncated and full-length L1s. It also biases against detection of all expressed L1 loci because the youngest L1 elements exhibit a high level of sequence similarity compared to older L1s (34,76,77). Similar to other alignment strategies, expression of polymorphic L1s is not detected under these conditions because these elements are not annotated in the mouse genome. To detect the expression of younger and non-reference human L1s, we have previously used a 5′ RACE/PacBio approach (36), which demonstrated that L1Hs loci also exhibit cell line specific expression. The 5′ UTR of the mouse and rat L1 contains tandem monomers, which are not friendly to the 5′RACE approach and would easily create chimeric products during PCR amplification. In our bioinformatics analyses we used strand-specific, paired-end reads and required concordant alignments in the sense direction with the corresponding L1 locus as previously described (34,36). This confirmed that transcripts occurred in the same orientation as the locus from which they originated and that transcripts were not spliced or otherwise processed.

The second key element in our strategy was the inspection of each potentially expressed L1 locus in the Integrated Genome Viewer (IGV) (78–80) to confirm that the transcript originated from the L1 promoter (Supplementary Figure S2 A–C). The importance of authenticating L1 loci expression via manual curation is shown in Supplementary Table S1 which demonstrates that, on average, only 5.6% of full-length L1 loci with uniquely aligned reads were scored as originating from the L1 promoter. The rest were due to variants of passive expression associated with other genes (34). The number of reads mapping to authentically expressed L1 loci were then normalized using FPKM.

To determine the number of reads corresponding to the youngest mouse L1 subfamilies that were discarded during our alignment, RNA-seq reads derived from RNA extracted from testes of 7.6 month old mice that were discarded during the Bowtie alignment step because they did not align uniquely to the mm10 genome were aligned to the active L1 mm10 subfamilies: T_f_, G_f_, and A (14). We found that an average of 317 read pairs (0.0011%), 143 read pairs (0.0005%), and 319 read pairs (0.0011%) of all discarded reads align to the promoter sequences of T_f_, G_f_, and A subfamilies, respectively. These reads correspond to both the L1 loci of T_f_, G_f_, and A subfamilies that were identified by our unique alignment and those that were not identified due to the lack of unique mappability.

### Mouse testes exhibit high total levels of L1 mRNA expression and high numbers of expressed L1 loci

We used mice as our experimental model to determine patterns of individual L1 locus expression *in vivo*. For the initial analysis we harvested cytoplasmic RNA from three testes, three livers, three brains and two lungs of 7.6 mo male mice to perform paired-end RNA sequencing. An overview of animals used in this study is shown in Supplementary Figure S3. RNA-Seq reads were aligned to the mm10 genome using stringent Bowtie alignment parameters, which include the exclusion of non-unique alignments, and manual curation of full-length L1 loci was performed as previously described (35,36). We normalized expression levels of authenticated L1 loci by calculating the FPKM for each locus as described in Materials and Methods. A complete list of expressed L1 loci and corresponding FPKM values per organ is provided in Supplementary Table S2.

**Figure 1.**
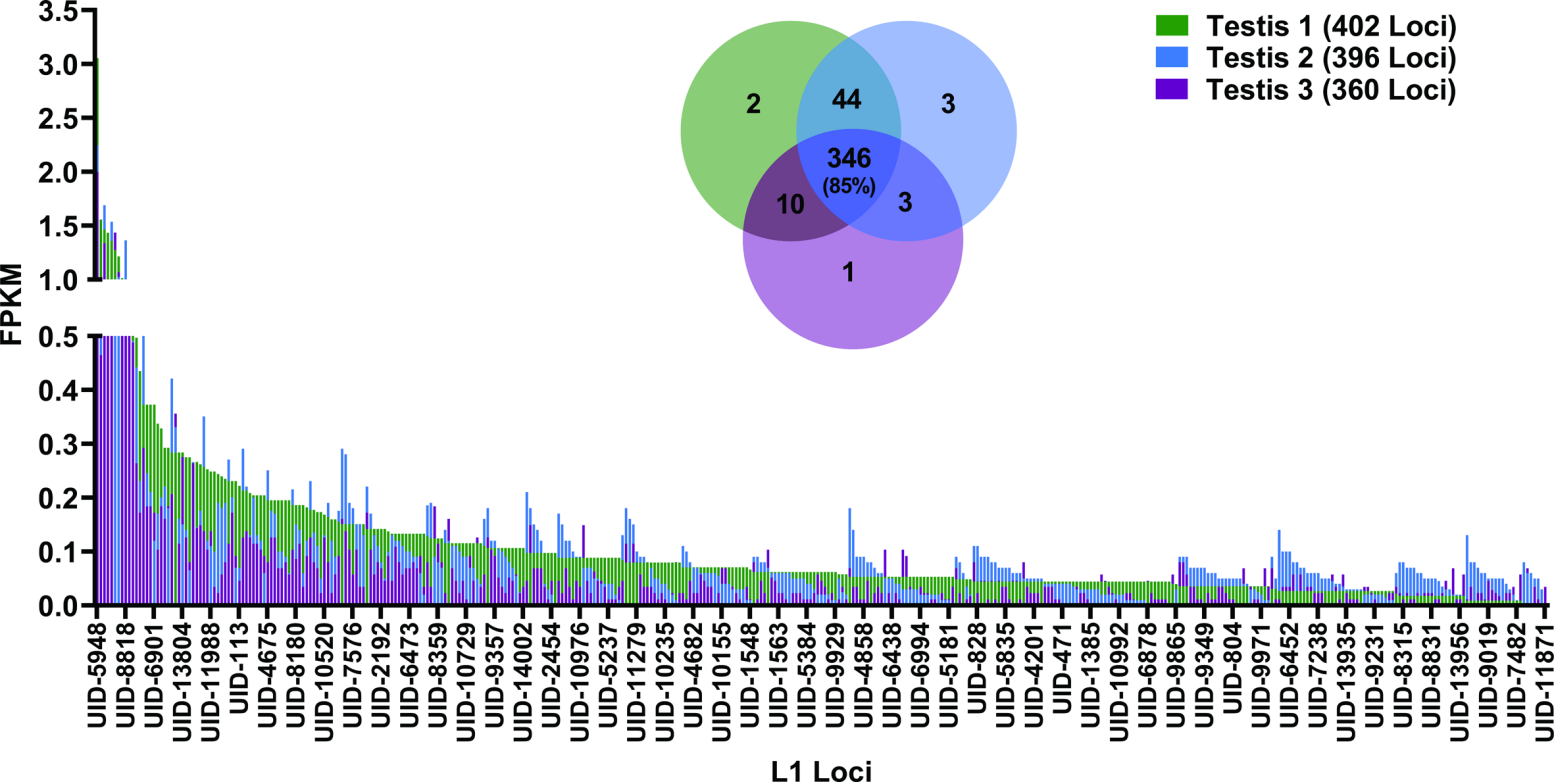
L1 mRNA expression in mouse testes. In the bar graph, expression levels of curated L1 mRNA-expressing loci in 7.6 mo mouse testes are quantified by FPKM. Analysis for each of three separate mice is presented as different colors. The total number of L1 mRNA-expressing loci in each of the three mouse testis samples are indicated in the graph key. The Venn diagram quantifies the number of shared loci between the three testis samples. The percent overlap is indicated in the center of the diagram.

**Figure 2.**
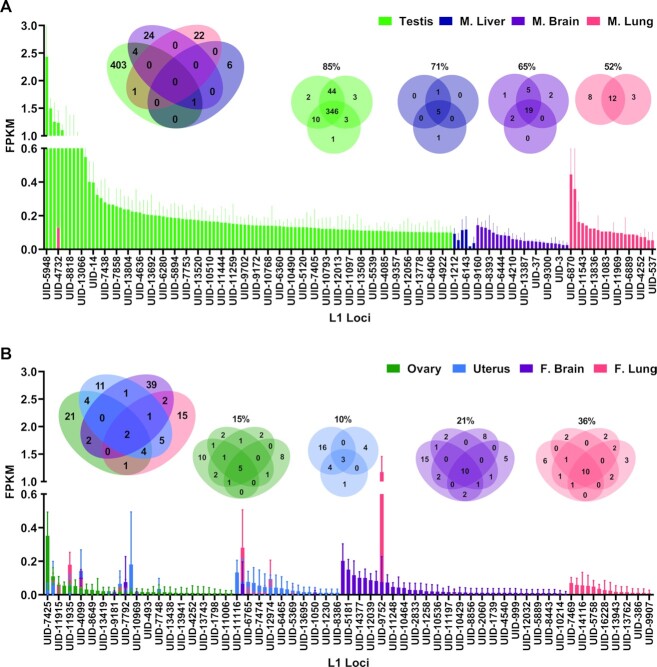
L1 mRNA expression in male and female organs. (**A**) A bar graph shows L1 mRNA expression levels of individual L1 loci that were identified to be expressed in different organs collected from 7.6 mo male mice. Expressed L1 loci are averaged for three testis samples, three liver samples, three brain samples and two lung samples. Expression from each organ is indicated in different colors with error bars representing standard deviation. Each expressed locus is listed only once on the X axis and loci expressed in multiple organs have overlapping FPKM bars. Due to the large number of expressed L1 loci, only some names are displayed on the X axis and a complete list of loci with corresponding FPKM values is in Supplementary Table S2. The Venn diagram (left) shows the overlap of expressed L1 loci between the male organs. The Venn diagrams (right) show the overlap of expressed L1 loci between samples for each organ collected from different mice. The percent overlap between the samples is indicated above each Venn diagram. (**B**) The bar graph represents L1 mRNA expression levels of L1 loci identified to be expressed in different organs collected from 8 to 8.7 mo female mice. Expressed L1 loci are indicated for four ovary samples, three uterus samples, four brain samples and four lung samples in different colors. Each expressed locus is listed only once on the X axis and loci expressed in multiple organs have overlapping FPKM bars. Due to the large number of expressed L1 loci, only some names are displayed on the X axis and a complete list of loci with corresponding FPKM values is in Supplementary Table S2. The Venn diagram (left) shows the overlap of expressed L1 loci between the female organs. The Venn diagrams (right) show the overlap of expressed L1 loci between samples for each organ. The percent overlap between the samples is indicated above each Venn diagram.

This analysis determined that adult mouse testes expressed a significantly higher number of L1 loci (*t*-test, *P* ≤ .005) and expressed 11 L1 loci at a higher level than any locus from the other organs (Figures 1 and 2A, Supplementary Figure S4A–C). Testes expressed an average of 386 L1 loci (Figure 1) above our mapped reads cutoff level, compared to an average of ∼6 L1 loci expressed in livers (Figure 2A, Supplementary Figure S4A), 25 L1 loci expressed in brains (Supplementary Figure S4B), and 18 L1 loci expressed in lungs (Supplementary Figure S4C). Livers expressed significantly fewer L1 loci than brains and lungs (*t*-test, *P* ≤ 0.05 and *P* ≤ 0.01, respectively). Testes also exhibited higher levels of expression at individual L1 loci. For example, L1 locus UID-5948 (UID, unique identifier) had the highest expression in testes with an FPKM of 3.05. In livers, the highest expressed L1 locus was UID-6143 with a 0.037 FPKM. In brains, the highest expressed L1 locus was UID-5181 with a 0.24 FPKM. In lungs, the highest expressed L1 locus was UID-6870 with a 0.83 FPKM. These data demonstrate that there is as much as a 100-fold difference in the expression levels of the highest expressed L1 loci among male organs of the same individual mouse, with testes being the highest and liver supporting the lowest levels of expression (Figure 2A).

**Figure 3.**
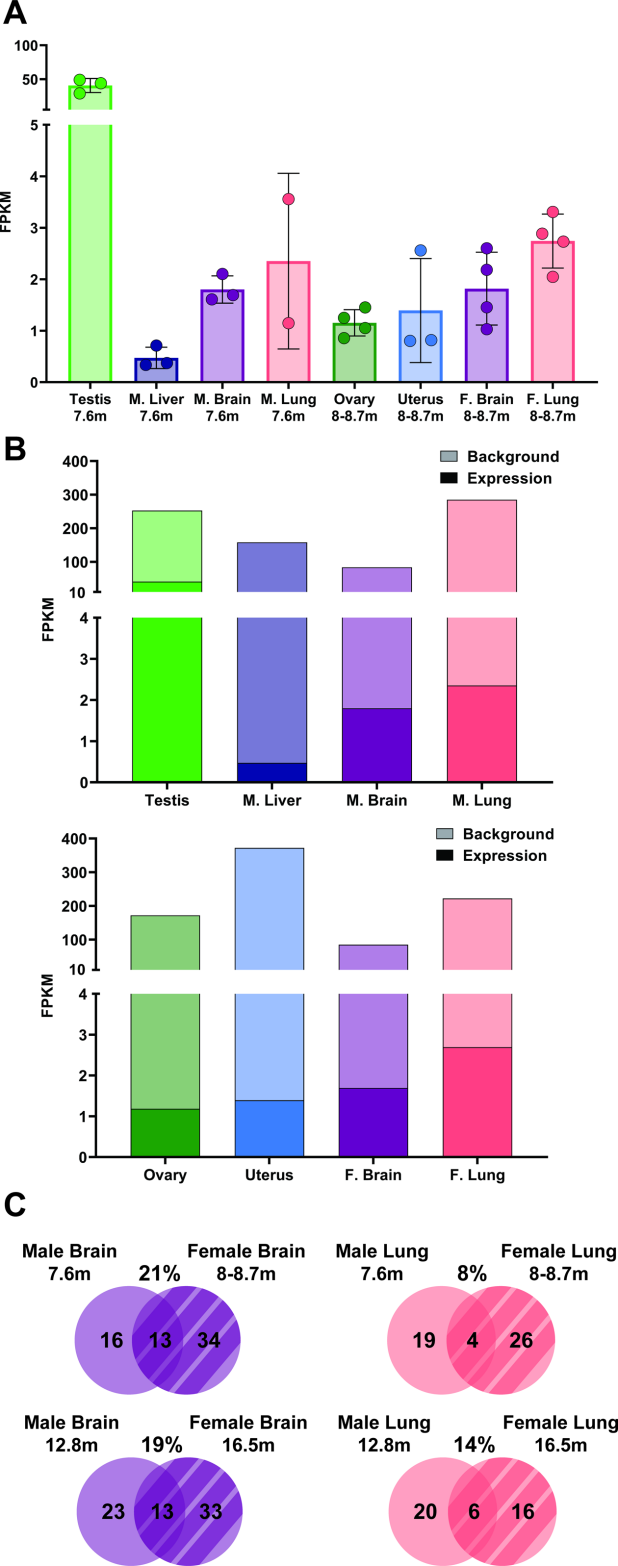
Background L1 expression is significantly higher than L1 mRNA expression. (**A**) Total L1 mRNA expression summing all expressed loci in organs collected from 7.6 mo male mice or 8–8.7 mo female mice. (**B**) L1 mRNA expression levels quantified by FPKM plotted in front of the FPKM values from background L1 sequences identified by RNA-Seq in males (top) and females (bottom). Bars with darker color represent the level of L1 mRNA expression and bars with lighter color represent background L1 sequences included in cellular RNAs. (**C**) In Venn diagrams, the overlap of expressed L1 loci between male and female organs are shown. L1 loci shared between male and female brains (left, purple) are quantified for mice 7.6–8.7 mo (top) and 12.8–16.5 mo (bottom). L1 loci shared between male and female lungs (right, pink) are quantified for the same age groups. The percent overlap is indicated above each diagram.

Using cytoplasmic RNA, we next analyzed L1 mRNA expression in four ovaries, three uteri, four brains and four lungs collected from 8 to 8.7 mo female mice (Supplementary Figure S3). This analysis demonstrated that no single female organ displayed a significant enrichment of expressed L1 loci comparable to the observations in testes. Ovaries expressed 34 L1 loci, on average. The highest expressed L1 locus in this organ, UID-7425, had a 0.5 FPKM (Figure 2B), which is 6 times lower than the highest expressed L1 locus in testes. Uteri expressed 28 L1 loci, on average. The highest expressed L1 locus in this organ, UID-7523, also had a 0.5 FPKM (Figure 2B). Female brains expressed 47 loci, on average. The highest expressed L1 locus in this organ, UID-869, had a 0.4 FPKM (Figure 2B), which is 1.7 times higher than the highest expressed L1 locus in male brains. Female lungs expressed 31 L1 loci, on average. The highest expressed L1 locus in this organ, UID-9752, had a 1.4 FPKM (Figure 2B), which is 2.3 times higher than the highest express L1 locus in male lungs. These moderate L1 expression levels observed in female organs are comparable with expression levels in corresponding male organs, apart from testes (Figure 3A). Of note, testis expressed significantly higher overall levels of L1 mRNA than any of the other organs analyzed (*t*-test, testis versus male liver, *P* = 0.0026, testis versus male brain, *P* = 0.0029, testis versus male lung, *P* = 0.0161, testis versus ovary, *P* = 0.0005, testis versus uterus, *P* = 0.0029, testis versus female brain, *P* = 0.0006, testis versus female lung, *P* = 0.0006) (Figure 3A).

### Patterns of L1 loci expression is organ-specific in mice

We compared specific expressed L1 loci between male organs. We found substantial overlap in expressed L1 loci between the same organs collected from different mice. Among all organs analyzed in this study, testis exhibited the greatest degree of similarity with 85% of testes loci (346 loci) shared between all three animals (Figures 1 and 2A). Liver samples expressed 5 (71%) shared L1 loci, brain samples expressed 19 (65%) shared L1 loci, and lung samples expressed 12 (52%) shared L1 loci (Figure 2A, Supplementary Figure S4 A–C).

In contrast to this high number of shared L1 loci expressed in the same organs from different individuals, there was minimal overlap between organs collected from the same male mouse. Of the combined 461 L1 loci expressed by all male organs considered, none (0%) were shared by all four organs in any of the mice, four (0.8%) were shared between testis and brain in six mice, 1 (0.2%) was shared between testis and lung in five mice and 1 (0.2%) was shared between testis, brain and liver in six mice (Figure 2A).

Female organs showed greater heterogeneity of expressed L1 loci than male organs. Of the 34 L1 loci expressed in ovaries, only five L1 loci (15%) were shared by all four samples. Of the 28 L1 loci expressed in uteri, only three loci (10%) were shared by all three samples. Of the 47 L1 loci expressed in female brains, 10 loci (21%) were shared by all four samples. Of the 30 L1 loci expressed in female lungs, 10 (36%) were shared by all four samples (Figure 2B). Similar to males, rather strict organ-specific patterns of L1 loci expression were observed in females even though 4 times fewer total expressed L1 loci were identified in females. Of the 108 L1 loci expressed by all female organs, only two loci (1.8%) were shared by all four organs considered in our analysis (Figure 2B).

Expression of L1 subfamilies also exhibit organ specificity in male and female mice (Supplementary Figure S9A). Testes have the highest percentage of the G_f_ and T_f_ subfamilies, the youngest and most active of the mouse L1 subfamilies (13,27,29,81). The percentage of expressed L1 loci in testes belonging to the G_f_ subfamily was significantly higher than livers, male brains, male lungs, ovaries, uteri, female brains, and female lungs (*t*-test, *P* < 0.0001, *P* < 0.0001, *P* = 0.0041, *P* < 0.0001, *P* = 0.0367, *P* = 0.0077, *P* = 0.0205, respectively). The percentage of expressed L1 loci in testes belonging to the T_f_ subfamily was significantly higher than livers, male lungs, ovaries, female brains and female lungs (*t*-test, *P* < 0.0001, *P* = 0.0034, *P* = 0.0038, *P* = 0.0020, *P* < 0.0001, respectively). The higher numbers of expressed young L1 subfamily members in testes indicate that loci expressed in the testes are more likely to participate in active retrotransposition (14).

Monomers in the 5′ UTR of mouse L1 elements function as promoters and increasing the number of monomers has been shown to increase reporter gene transcription (25,82). Analysis of the number of monomers in expressed L1 loci for each subfamily per organ did not detect any significant difference between organs and identified that ∼2–4 monomers are sufficient for L1 mRNA expression (Supplementary Figure S9B).

### Background L1 sequences unrelated to L1 mRNA expression are abundant and tissue specific

The central goal of our manual curation step in the bioinformatics pipeline is to filter out noise caused by L1 sequences generated through passive cellular transcription (34,36). Although passively expressed L1 sequences may affect cellular function, they do not contribute to L1 mRNAs that are related to the L1 retrotransposition cycle. During our analysis, we quantified the amount of these passive, background alignments caused by reads aligning to L1 sequences that did not represent L1 mRNA expression from its own promoter. We found that overall, 98% of aligned L1 reads were generated from passive cellular transcription. We demonstrate how the failure to filter out background noise by using conventional techniques such as RT-PCR or non-customized bioinformatics analyses would affect our results by overlaying the authentic expression levels in dark colors over the background levels in faded colors (Figure 3B). Comparing background levels alone between organs skews the results such that uteri and male lungs would have the highest levels of total L1 expression. Authentic L1 mRNA expression accounted for 13.9% of the total FPKM value in testes, 0.3% in livers, 2.1% in male brains, 0.8% in male lungs, 0.7% in ovaries, 0.4% in uteri, 1.9% in female brains and 1.2% in female lungs (Figure 3B, Supplementary Figure S5C).

Next, we plotted expression levels for L1 loci identified as background that have FPKM levels >0.1 in males and females (Supplementary Figure S5A and B). These graphs show a considerable degree of overlap between organs and high expression levels from individual loci, compared to L1 mRNA expression levels shown in Figure 2A and B. All male organs shared 11 (5%) background L1 loci and all female organs shared 26 (19%) background L1 loci (Supplementary Figure S5A and B), which were higher degrees of similarity compared to the shared authentically expressed L1 loci in male (0%) and female (1.8%) organs (Figure 2A and B). L1 loci UID-3342 and UID-13571 had the highest background FPKM values in every organ. Both UID-3342 and UID-13571 were classified as background because they had a large pile up of reads that did not correlate with the ‘mappability’ of the L1 locus (36).

### L1 mRNA expression in mouse organs exhibits longitudinal changes in an organ- and sex-specific manner

**Figure 4.**
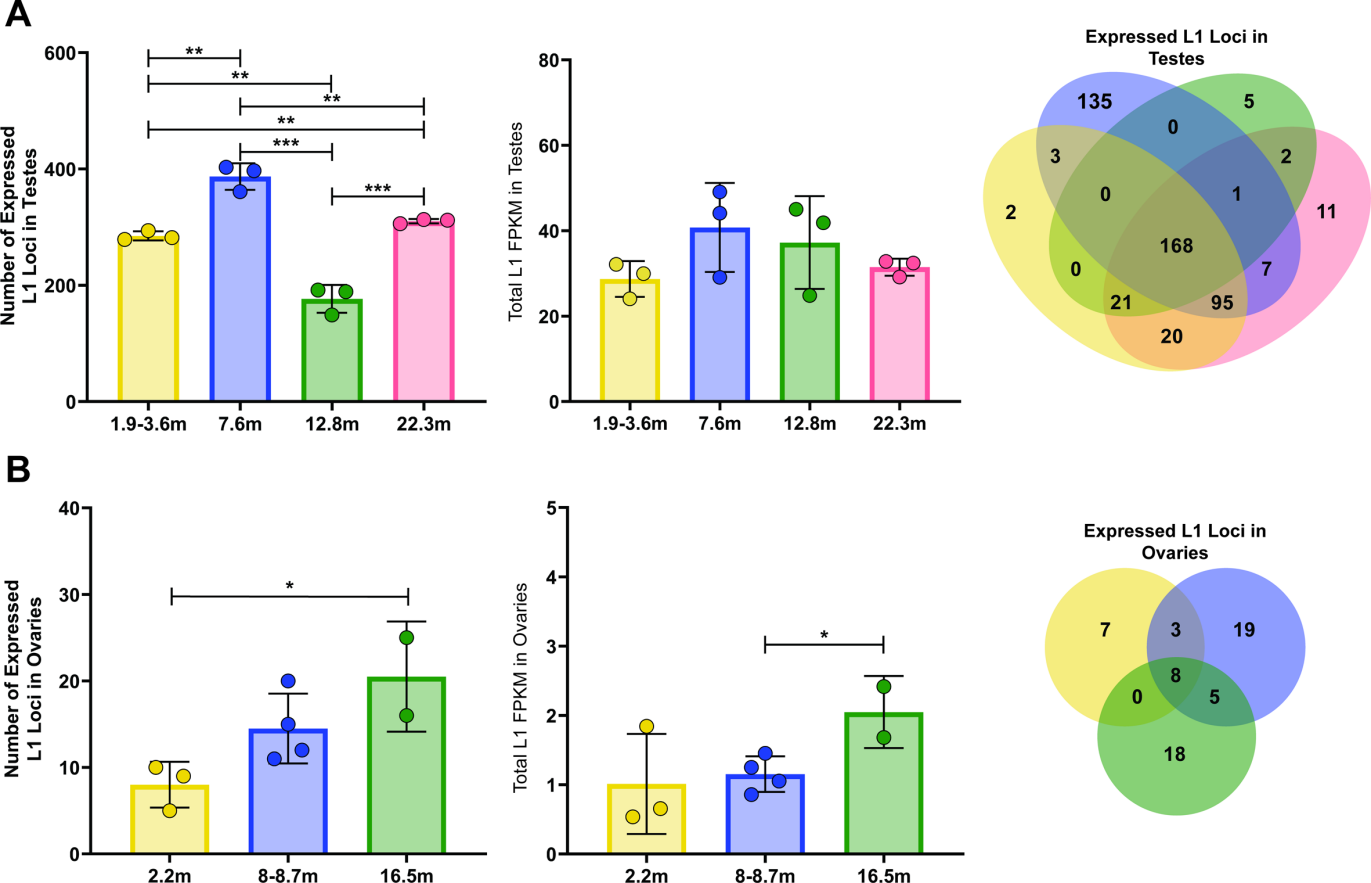
L1 mRNA expression exhibits different patterns of fluctuation with age in male and female organs. (**A**) Pattern of L1 mRNA expression in testes collected from mice of four different age groups. Left to right: the graph on left shows the number of L1 loci expressed in testes collected from mice 1.9–3.6, 7.6, 12.8 or 22.3 mo. Three mice were used for each age group. Filled circles correspond to values determined for an individual mouse. Filled bars represent mean number of expressed L1 loci in each age group. Error bars show standard deviations (*t*-test, *** ≤ 0.005, ** ≤ 0.01, * ≤ 0.05). The middle graph shows the total L1 mRNA expression level for each group determined by summing levels of all expressed L1 loci measured by FPKM for each sample. The Venn diagram shows overlap of expressed L1 loci shared between different age groups with 168 L1 loci being expressed in all age groups. (**B**) Pattern of L1 expression by age in ovaries. Left to right, the number of expressed L1 loci in ovaries collected from mice 2.2, 8–8.7 or 16.5 mo (*t*-test, *** ≤ 0.005, ** ≤ 0.01, * ≤ 0.05). The total L1 mRNA expression level is shown as FPKM for each sample (*t*-test, *** ≤ 0.005, ** ≤ 0.01, * ≤ 0.05). The Venn diagram shows overlap of expressed L1 loci between different age groups, with eight L1 loci being expressed in all three age groups.

Normal mammalian aging is associated with transcriptional deregulation often arising from intrinsic changes and extrinsic exposures (60,83). To determine whether there are any longitudinal changes in L1 mRNA expression at a single-locus resolution we analyzed male and female mouse organs collected at ages ranging from 1.9 to 22.3 months. In testes, we observed significant changes in the number of L1 loci expressed between different age groups (Figure 4A). Even though there was no significant difference in the total levels of L1 mRNA expression between these age groups (Figure 4), there was a significant increase in the number of loci (increased by 135) between the 1.9–3.6 and 7.6 mo groups (*t*-test, *P* = 0.0018). The 135 L1 loci uniquely expressed in the 7.6 mo group accounted for 8.57 FPKM (20.6%) of the 41.56 FPKM for total L1 expression observed at 7.6 months (Figure 4A). The average number of expressed L1 loci was significantly lower in the 12.8 mo group compared to the 7.6 mo group with an average of 210 fewer expressed L1 loci in the 12.8 mo group, again without any difference in the total levels of L1 mRNA expression between these age groups (*t*-test, *P* = 0.0004). 22.3 mo testes expressed significantly more L1 loci on average than the 12.8 mo group with the 22.3 mo group expressing on average 134 more L1 loci than the 12.8 mo group (*t*-test, *P* = 0.0007). Of the 471 L1 loci expressed in testis of all age groups, 168 (36%) were shared between all age groups (Figure 4A, Supplementary Figure S6A3, B3, C3, D3, E3, F3, Supplementary Table S3). These findings demonstrate that significant changes in patterns of L1 loci expression occur during aging. They also show that analysis of total levels of L1 mRNA expression alone is not enough to fully understand longitudinal dynamics in L1 loci expression and their potential contribution to the aging process.

Male livers exhibited an age-associated pattern in expressed L1 loci similar to that observed in testes with 7.6 mo livers expressing significantly more L1 loci than 1.9–3.6 mo livers, 12.8 mo livers and 22.3 mo livers (*t*-test, *P* = 0.0002, *P* = 0.0006 and *P* = 0.02, respectively; Supplementary Figure S6A1). Additionally, the 22.3 mo livers expressed significantly more L1 loci, compared to 1.9–3.6 mo livers and 12.8 mo livers (*t*-test, *P* = 0.003 and *P* = 0.01, respectively). Although a low number of expressed L1 loci was detected in livers collected from male mice of all age groups, livers collected from 7.6 mo male mice expressed an average of four more L1 loci, compared to the 1.9–3.6 mo age group (*t*-test, *P* = 0.0002). 12.8 mo livers expressed on average three fewer L1 loci than the 7.6 mo age group (*t*-test, *P* = 0.0006). 22.3 mo livers expressed on average two more L1 loci than the 12.8 mo age group (*t*-test, *P* = 0.01) and three more than the 1.9–3.6 mo age group (*t*-test, *P* = 0.003, Supplementary Figure S6A2). Male brains had no significant change in the number of expressed L1 loci between the 7.6 and 12.8 mo groups. However, male brains did exhibit a significant increase in total L1 mRNA expression with the 7.6 mo group averaging a 1.8 FPKM and the 12.8 mo group averaging a 3.65 FPKM across all expressed L1 loci (*t*-test, *P* = .02, Supplementary Figure S6B1 and B2, Supplementary Figure S7). Male lungs showed a marginally significant increase in the number of expressed L1 loci with the 12.8 mo group expressing an average of 24 L1 loci and the 7.6 mo group expressing an average of 18 L1 loci (*t*-test, *P* = 0.05, Supplementary Figure S6 C1 and C2). Of note, the ages of 7.6 mo and 22.3 mo mice that demonstrate significantly higher numbers of L1 loci expressed in testis correspond to 38–47-year-old humans and >65-year-old humans, respectively (84).

We next investigated the effect of aging on the number of expressed L1s and the total level of L1 mRNA expression in organs of 2.2, 8–8.7 and 16.5 mo female mice. In ovaries, the oldest age group, 16.5 mo, expressed on average 13 more L1 loci than the 2.2 mo group (*t*-test, *P* = 0.05, Figure 4B). The 16.5 mo group also averaged 2.05 FPKM for total L1 expression, which was 0.89 FPKM greater than the 8–8.7 mo group (*t*-test, *P* = 0.04, Figure 4B, Supplementary Figure S7). This analysis demonstrated a trend in which the number of L1 loci and total L1 mRNA expression increases in ovaries in the oldest mouse age group, which corresponds to 50–56-year-old humans (84). Uteri and female lungs did not show significant changes in either the number of expressed L1 loci or the total level of L1 mRNA expression with age (Supplementary Figure S6D1, D2, F1 and F2). Female brains exhibited a trend similar to the one observed in ovaries, i.e. the 2.2 and 16.5 mo groups significantly differed in the number of expressed L1 loci. 2.2 mo brains expressed an average of ∼14 L1 loci while 16.5 mo brains expressed 32 L1 loci (*t*-test, *P* = 0.01). In addition to the increase in the number of expressed L1 loci in brains of older female mice, total levels of L1 expression also increased from an average of 1.25 FPKM in the 2.2 mo group to 3.47 FPKM in the oldest age group (*t*-test, *P* = 0.0254, Supplementary Figure S6E1 and E2, Supplementary Figure S7). Overall, these findings demonstrate that the number of expressed L1 loci and total L1 mRNA expression levels change as independent variables with age in an organ- and sex-specific manner.

To further investigate effects of age on L1 loci expression, we quantified the number of shared L1 loci between different age groups by organ. Ovaries and uteri displayed the greatest amount of variation between age groups (Supplementary Table S3). Ovaries from different age groups displayed a 13% overlap between all age groups and uteri from different age groups displayed a 2.2% overlap between all age groups. We hypothesize that this low level of overlap in expressed L1 loci in female reproductive organs could be due to hormonal fluctuation, leading to changes in transcription (84,85). Analysis of mouse mRNA expression in uteri samples across all age groups using EBSeq found that one of the uterus samples, labeled as 8–8.7m 1 in Supplementary Figure S8, expressed higher levels of *wif1* and *lgr5* genes, expression of which indicates that a mouse uterus is in the estrus phase (72,85). The 8–8.7 m 2 and 3 uterus samples expressed higher levels of the *syt16* and *tmprss11e* genes, expression of which indicates that a mouse uterus is in the proestrus phase (Supplementary Figure S8) (85). This difference provides a possible explanation for the observed variation in L1 loci expression between uteri in the 8 mo group as well as between age groups since the 16.5 mo samples are post-menopausal (85,86) (Supplementary Figure S8). We also observed that in ovaries, the 2.2 mo group had significantly fewer expressed L1 loci in common, compared to the 16.5 mo group (1 versus 10 loci, chi-square, *X*^2^ (1, *N* = 8) = 3.864, *P* = 0.05, Supplementary Table S3). This shows that ovaries share more expressed L1 loci at an older age, further indicating that changes in hormonal fluctuations that occur with age may influence variation in L1 mRNA and/or loci expression. Female brains and lungs share more L1 loci across different ages with 23% L1 loci overlap in all brain age groups and 32% overlap in all lung age groups (Supplementary Table S3). In males, testes display a high level of consistency with 36% L1 loci shared between all age groups (Supplementary Table S3). Male liver, brain, and lung share fewer expressed L1 loci through age than testes with 9% shared in livers, 23% shared in brains, and 9% shared in lungs (Supplementary Table S3). Testes samples collected from mice of different ages share significantly fewer expressed L1 loci, a pattern opposite to that observed in ovaries. 1.9–3.6 mo testes share significantly more expressed L1 loci than 22.3 mo testes (chi-square, *X*^2^(1, *N* = 6) = 8.8147, *P* = 0.0029) (Supplementary Table S3). 7.6 mo testes share significantly more expressed L1 loci than 12.8 mo testes (chi-square, *X*^2^(1, *N* = 8) = 8.809, *P* = 0.0024, Supplementary Table S3). 12.8 mo testes share significantly more expressed L1 loci than 22.3 mo testes (chi-square, *X*^2^(1, *N* = 6) = 20.219, *p* < 0.00001) (Supplementary Table S3).

### Sexual dimorphism of L1 loci expression in mice

We have demonstrated that L1 loci are expressed in an organ-specific manner in both male and female mice (Figure 2). To determine whether expression of L1 loci also exhibits sex-specific patterns, we compared the number of shared L1 loci between all male and female organs. We found that male and female mice only share 37 (5%) of the combined 690 L1 loci expressed in males and females of all age groups (Supplementary Table S3, sheet Male v Female, lines 1–3). Breaking down the number of shared loci by age and sex, we observed that of the 59 L1 loci expressed in the 2.2 mo female age group and the 312 L1 loci expressed in the 1.9–3.6 mo male age group, 2 (0.5%) expressed L1 loci are shared between male and female organs. The 2 shared L1 loci represent 3.4% and 0.6% of the total L1 loci expressed in females and males of this age group, respectively (Supplementary Table S3). Of the 108 L1 loci expressed in the 8–8.7 mo female age group and the 461 L1 loci expressed in the 7.6 mo male age group, 21 (3.7% of total L1 loci in these groups) expressed L1 loci are shared between male and female organs. Of the 104 L1 loci expressed in the 16.5 mo female age group and the 256 L1 loci expressed in the 12.8 mo male age group, 21 (6.2% of the total L1 loci in these groups) L1 loci are shared. The 21 shared L1 loci represent 19% and 4.5% of the total L1 loci expressed in females and males of the 8–8.7 and 7.6 mo age groups, respectively, and 20% and 8.2% of the total L1 loci expressed in females and males of the 16.5- and 12.8 mo age groups, respectively. Statistical analysis determined that the 21 expressed L1 loci shared between the 8 and 8.7 mo females and the 7.6 mo males is significantly greater than the two expressed L1 loci shared between the 2.2 mo female age group and the 1.9–3.6 mo male age group (chi-square, X^2^(1,N = 13) = 9.9157, *P* = .00703). Similarly, the 21 expressed L1 loci shared between the 16.5 mo female age group and the 12.8 mo male age group is significantly greater than the two expressed L1 loci shared between the 2.2 mo female age group and the 1.9–3.6 mo male age group (chi-square, *X*^2^(1,*N* = 11) = 29.6273, *P* < 0.00001). Taken together, this finding demonstrates that L1 loci expression is sex-specific and remains so with age even though there is a 10-fold (2–21 loci) increase in the number of shared L1 loci between sexes with age.

We compared expression patterns of L1 loci between male and female organs of the same type and age group to better understand L1 mRNA expression in different sexes. Lungs collected from 8–8.7 mo females and 7.6 mo males expressed 30 and 23 L1 loci, respectively, and shared 4 (8%) expressed L1 loci (Figure 3C and Supplementary Table S3, sheet Male v Female, line 19). In comparison, lungs collected from female mice in this age group shared 10 (33%) L1 loci with each other (Supplementary Table S3, Aging, line 5, Supplementary Figure S7 H2) and male mice of this age group shared 12 (52%) L1 loci with each other (Supplementary Table S3, Aging, line 12, Supplementary Figure S7 D1). By performing a chi-squared test, we determined that significantly fewer expressed L1 loci were shared between male and female lungs in these groups than the number of L1 loci shared among lungs collected from either males or females of these respective age groups (males chi square, *X*^2^(1, *N* = 5) = 7.311, *P* = 0.007, females chi square, *X*^2^(1, *N* = 5) = 16.27, *P* = 0.00006, Supplementary Table S3, sheet Male v Female, lines 23–24). Similarly, lungs collected from 16.5 mo females and 12.8 mo males expressed 22 and 26 L1 loci, respectively, and shared 6 (14%) expressed L1 loci (Figure 3C and Supplementary table S3, Male v Female, lines 16–20). In comparison, the lungs collected from female mice in this age group shared 3 (14%) L1 loci with each other (Supplementary Table S3, Aging, line 6, Supplementary Figure S7 H3) and male mice of this age group shared 21 (81%) L1 loci with each other (Supplementary Table S3, Aging, line 13, Supplementary Figure S7 D2). We determined that significantly fewer L1 loci are shared between male and female lungs in these age groups, compared to L1 loci shared among lungs collected from males (chi square, *X*^2^ (1, *N* = 5) = 33.9183, *P* < 0.00001, Supplementary Table S3, Male v Female, line 26). The number of shared L1 loci between male and female lungs did not significantly differ from the number of L1 loci shared among lungs collected from females in the 16.5 mo age group. Taken together, these results show that in terms of expressed L1 loci, in general lungs are more similar within the same sex than between sexes except in 16.5 mo females.

We also compared expressed L1 loci between male and female brains at different ages. Brains collected from 8 to 8.7 mo females and 7.6 mo males expressed 47 and 29 L1 loci, respectively, and shared 13 (21%) expressed L1 loci (Figure 3C and Supplementary table S3, Male v Female, line 30). In comparison, brains collected from female mice in this age group shared 10 (21%) L1 loci with one another (Supplementary Table S3, Aging, line 5, Supplementary Figure S7 G2) and male mice of this age group shared 19 (65%) L1 loci with each other (Supplementary Table S3, Aging, line 12, Supplementary Figure S7 C1). We found that significantly fewer L1 loci are shared between male and female brains in this age group, compared to L1 loci shared among brains collected from males of this age group (chi square, X^2^ (1, *N* = 5) = 23.2185, *P* < 0.00001, Supplementary Table S3, Male v Female, line 35), but not female brains (Supplementary Table S3, Male v Female, lines 34–35). Brains collected from 16.5 mo females and 12.8 mo males expressed 46 and 36 L1 loci, respectively, and shared 13 (19%) expressed L1 loci (Figure 3C and Supplementary table S3, Male v Female, line 31). In comparison, brains collected from female mice in this age group shared 17 (37%) L1 loci with each other (Supplementary Table S3, Aging, lines 6, Supplementary Figure S7 C2) and male mice of this age group shared 17 (47%) L1 loci with each other (Supplementary Table S3, Aging, lines 14, Supplementary Figure S7 G3). We also found that significantly fewer L1 loci are shared between male and female brains in this age group, compared to L1 loci shared among brains collected from either males or females of these age groups (males chi square, *X*^2^(1, *N* = 5) = 12.9833, *P* < 0.0003, females chi square, *X*^2^(1, *N* = 5) = 7.3134, *P* = 0.007, Supplementary Table S3, Male v Female, lines 36–37). These data show that in terms of expressed L1 loci, brains of 8–8.7 mo females are as different from each other as they are from the 7.6 mo brains, while 12.8 male and 16.5 female brains are significantly more similar within the same sex compared to the opposite sex.

### Expressed mouse L1 loci are frequently found in expressed genes

**Figure 5.**
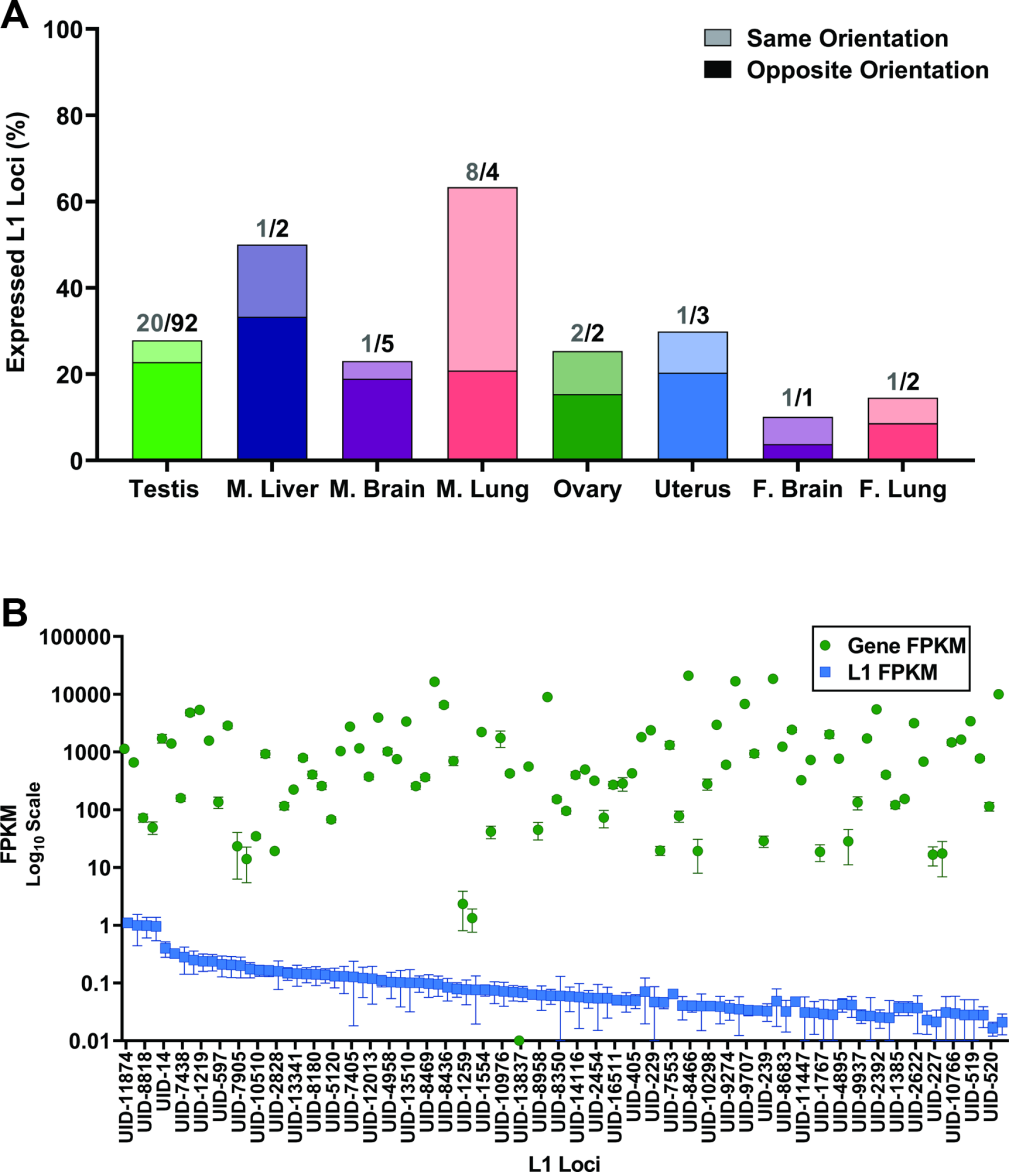
Expressed L1 loci are present in expressed and unexpressed genes. (**A**) Percent of expressed L1 loci that occur in genes. Light color represents the percent of expressed L1 loci that are found in genes in the same orientation as the gene. Darker color represents the percent of expressed L1 loci found to be in genes in the opposite orientation relative to the said gene. The numbers above each bar represent the number of L1 loci that were in genes of the same orientation (grey)/ the numbers of L1 loci that were in genes of the opposite orientation (black). (**B**) L1 FPKM levels for each expressed locus in a gene (blue squares) are plotted alongside the FPKM corresponding to the gene (green circles) in which the expressed L1 occurs.

Our analysis of L1 mRNA expression in mice identified hundreds of expressed L1 loci, allowing us to begin to understand what distinguishes L1 loci that are expressed from those that are not. We next investigated whether intragenic L1 loci are more likely to be expressed. First, by intersecting the genomic coordinates of expressed L1 loci with the known gene coordinates in the mm10 genome downloaded from the UCSC Genome Browser, we identified intragenic, expressed L1 loci and their orientation relative to genes (Figure 5). We found that 10–63% of expressed L1 loci per organ were within genes, all of which were in gene introns (Figure 5A). Male lungs had the highest number of expressed L1 loci that were also in genes (63%) and female brains had the fewest number of expressed L1 loci that were also in genes (10%). Female brains and female lungs contained an average of 10 and 15 expressed L1 loci that were in genes, respectively, which was significantly fewer than the average of 23 and 63 expressed L1 loci that were in genes in male brains and male lungs, respectively (*t*-test, brains, *P* = 0.0006; lungs, *P* = 0.0001).

Next, we considered whether expression of intragenic L1 loci correlated with expression of the genes in which they were present. We plotted L1 FPKM levels alongside the FPKM of their corresponding gene expressed in testes collected from 7.6 mo mice (Figure 5B). Although we did not find significant correlation between gene expression and L1 expression levels (Pearson correlation, *r*(92) = –0.1, *P* = 0.3), we did find that all genes, except one, that contain an expressed L1 locus are also expressed. Thus, gene expression provides a critical favorable environment, but does not determine L1 expression strength.

### Differential expression of RNA biogenesis pathways correlates with patterns of L1 mRNA expression

**Figure 6.**
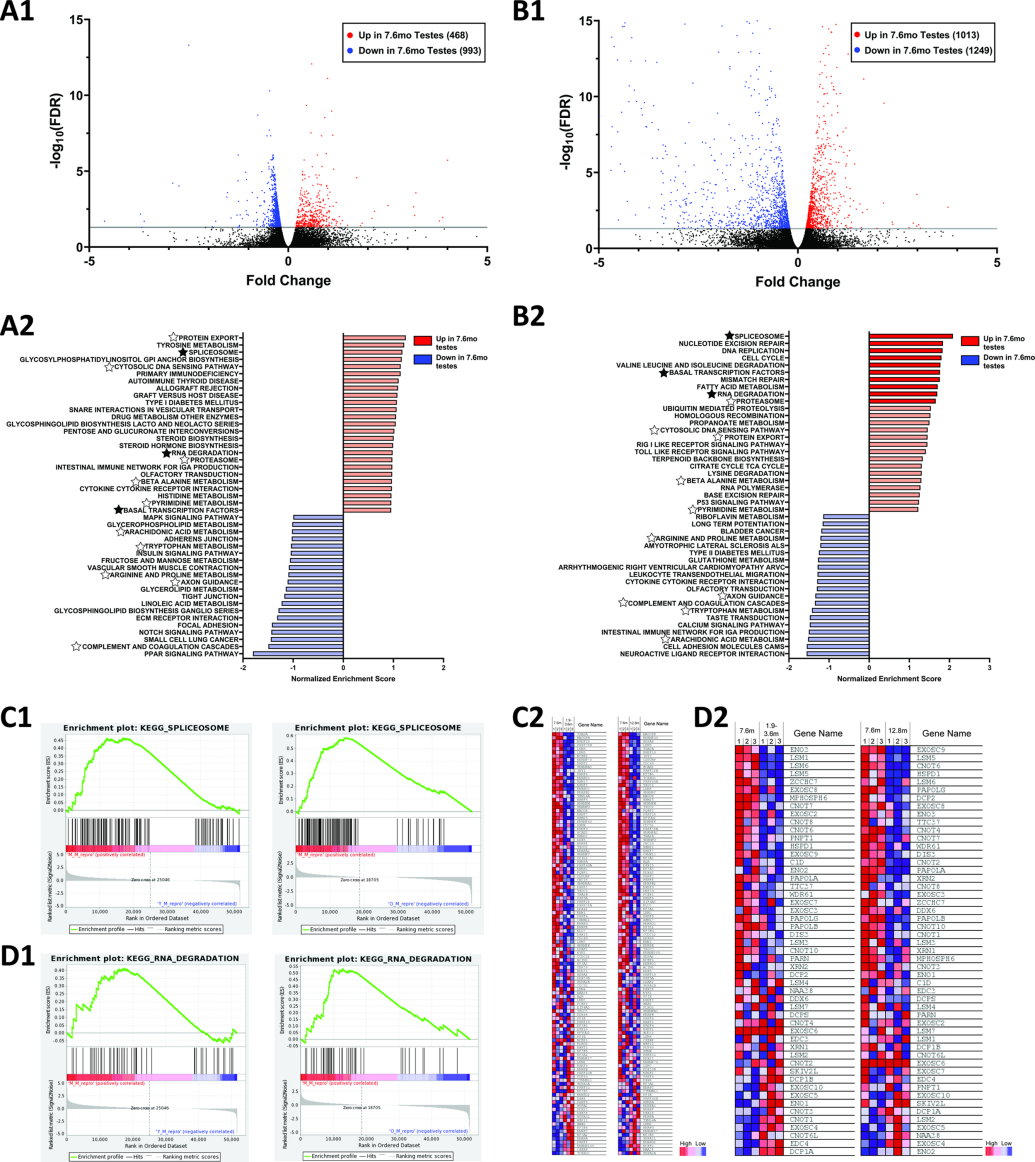
Transcriptomic profiling identifies differentially regulated pathways associated with L1 mRNA biogenesis. (**A1**) The volcano plot shows differentially expressed genes in 7.6 mo testes versus 1.9–3.6 mo testes. The grey line indicates FDR = 0.05. 468 genes are upregulated in 7.6 mo testes (red) and 993 genes are downregulated in 7.6 mo testes (blue), compared to 1.9–3.6 mo testes. 10 genes are not within axis limits. (**A2**) Gene set enrichment analysis showing the normalized enrichment score for the top 25 upregulated (red) and top 20 downregulated (blue) gene sets in 7.6 mo testes compared to 1.9–3.6 mo testes. Dark red or blue indicates a significant *p*-value (*P* < 0.05), faded red or blue indicates a nonsignificant *p*-value. Pathways common between A2 and B2 are indicated with a star, stars for pathways potentially linked to L1 mRNA expression and processing are filled. (**B1**) Differentially expressed genes in 7.6 mo testes versus 12.8 mo testes. The grey line indicates FDR = 0.05. 1013 genes are upregulated in 7.6 mo testes (red) and 1249 genes are downregulated in 7.6 mo testes (blue) compared to 1.9–3.6 mo testes. 103 genes are not within axis limits. (**B2**) Gene set enrichment analysis showing the normalized enrichment score for the top 25 upregulated (red) and top 20 downregulated (blue) gene sets in 7.6 mo testes compared to 12.3 mo testes. Dark red or blue indicates a significant *p*-value (*P* < 0.05), faded red or blue indicates a nonsignificant *P*-value. Pathways common between A2 and B2 are indicated with a star, stars for pathways linked to RNA biogenesis are filled. (**C1**) Spliceosome gene set enrichment plots for 7.6 mo testes compared to 1.9–3.6 mo testes (left) and 7.6 mo testes compared to 12.8 mo testes (right). (**C2**) Heatmaps for genes in the spliceosome pathway with comparisons between 7.6 mo and 1.9–3.6 mo testes are on the left and comparisons between 7.6 mo and 12.8 mo testes are on the right. (**D1**) RNA degradation gene set enrichment plots for 7.6 mo testes compared to 1.9–3.6 mo testes (left) and 7.6 mo testes compared to 12.8 mo testes (right). (**D2**) Heatmaps for genes in the RNA degradation pathway with comparisons between 7.6 mo and 1.9–3.6 mo testes are on the left and comparisons between 7.6 mo and 12.8 mo testes are on the right.

We performed transcriptomic profiling of our RNA-sequencing data to identify potential mechanisms contributing to the observed patterns of L1 mRNA expression in testis because mechanisms governing L1 mRNA expression are numerous and complex (58). We used Kallisto and Sleuth to measure and quantify differences in gene expression between 7.6 mo testes and both 1.9–3.6 mo testes and 12.8 mo testes, the three age groups that exhibited the most significant differences in the number of expressed L1 loci without significant changes in the total L1 mRNA expression (Figure 4A and B). This analysis identified that 7.6 mo testes have 1461 significantly differentially regulated genes compared to 1.9–3.6 mo testes (FDR < 0.05, 468 upregulated genes, 993 downregulated genes) (Figure 6A1) and 2262 genes compared to 12.8 mo testes (FDR < 0.05, 1013 upregulated genes, 1249 downregulated genes) (Figure 6B1). Gene set enrichment analysis identified several pathways related to DNA repair, metabolism, transcription, and RNA biogenesis among the top pathways to be upregulated in 7.6 mo testes compared to 1.9–3.6 mo testes and significantly upregulated compared to 12.8 mo testes (spliceosome FDR = 0.0026, RNA degradation FDR = 0.029, Basal transcription factors FDR = 0.021) (Figure 6, A2 and B2, filled stars). Upregulation of spliceosome, RNA degradation, and basal transcription factors pathways are relevant to our findings because these pathways are involved in RNA biogenesis and may play a role in the number of expressed L1 loci and L1 mRNA accumulation (9,87–90).

**Figure 7.**
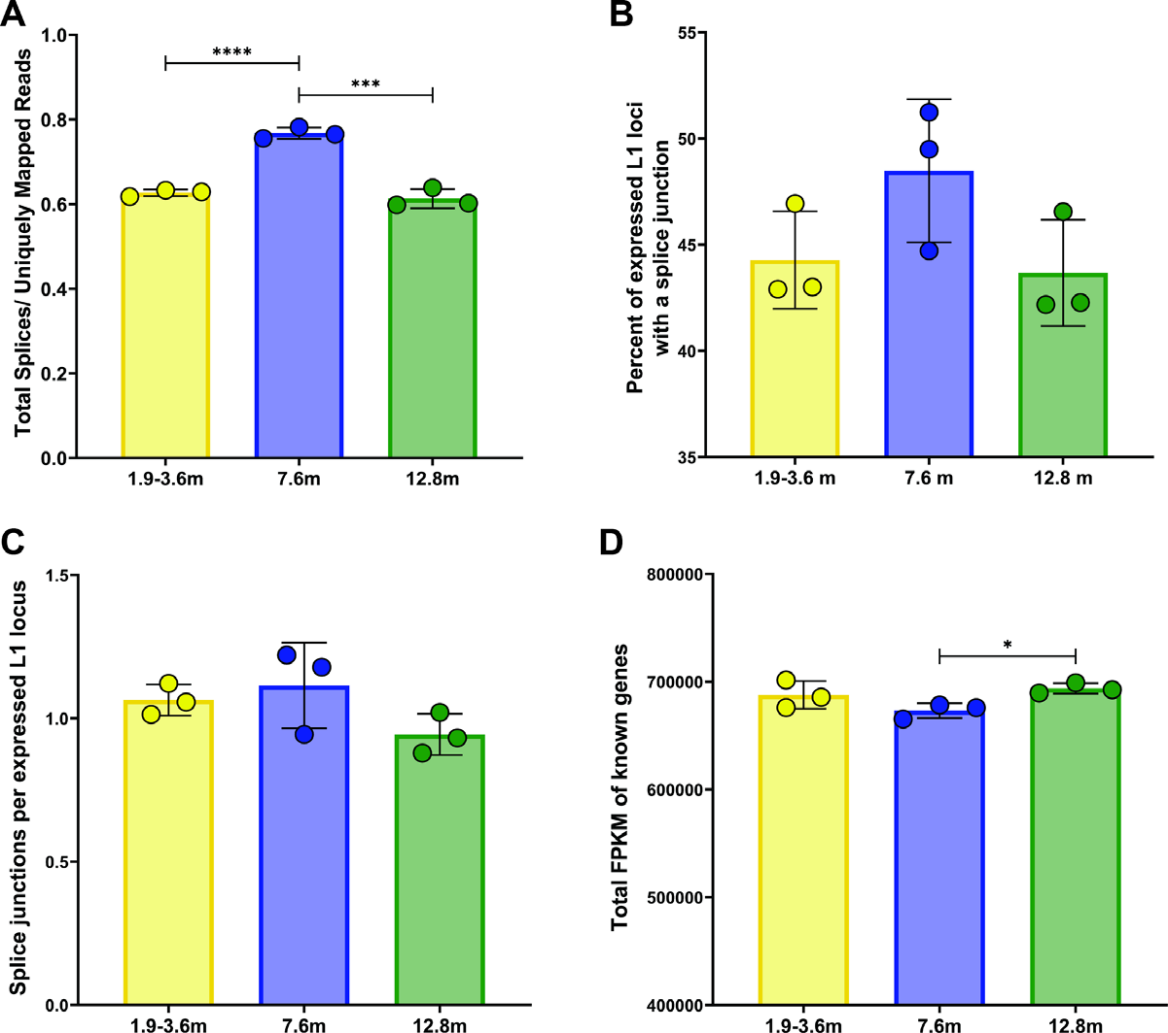
L1 mRNA splicing and total transcript levels fluctuate with age in testes. (**A**) Quantification of the total number of spliced reads for 1.9–3.6 mo testes, 7.6 mo testes, and 12.8 mo testes, normalized by the number of uniquely mapped reads per sample (*t*-test, **** < 0.0001, *** ≤ 0.005). (**B**) The percent of expressed L1 loci with at least one splice junction in 1.9–3.6 mo testes, 7.6 mo testes, and 12.8 mo testes. (**C**) A bar graph quantifying the number of splice junctions per expressed L1 locus in 1.9–3.6 mo testes, 7.6 mo testes, and 12.8 mo testes. (**D**) A bar graph quantifying the total mRNA levels of known genes in 1.9–3.6 mo testes, 7.6 mo testes and 12.8 mo testes, normalized by FPKM (*t*-test, * ≤ 0.05).

We identified upregulation of 62 of 116 spliceosome pathway genes in 7.6 mo testes compared to 1.9–3.6 mo testes and upregulation of 97 of 116 spliceosome pathway genes in 7.6 mo testes compared to 12.8 mo testes (Figure 6, C1, C2). Quantification of the number of splice events in testes from different age groups, calculated by running STAR and normalizing the total number of spliced reads to uniquely mapped reads, determined that 7.6 mo testes have significantly more total spliced reads compared to 1.9–3.6 mo testes (*t*-test, *P* < 0.0001) and 12.8 mo testes (*t*-test, *P* = 0.0005) (Figure 7A). Quantifying the percentage of expressed L1 loci with at least one splice junction (Figure 7B) and the number of splice junctions per expressed L1 locus (Figure 7C) revealed a trend of an increased number of splice events in L1 loci expressed in 7.6 mo testes, compared to 1.9–3.6 mo and 12.8 mo testes, although no significance was detected in these comparisons. The enrichment of splice events in 7.6 mo testes is consistent with upregulation of the spliceosome pathway (Figure 6), suggesting that increased splicing involving expressed L1 loci may contribute to the lack of increase in total L1 mRNA in the 7.6 mo age group despite the increase in the number of expressed L1 loci (Figure 4A and B). However, since these differences are modest, there are likely additional mechanisms contributing to this pattern of L1 mRNA expression.

We also observed upregulation of 35 of 51 RNA degradation pathway genes in 7.6 mo testes compared to 1.9–3.6 mo testes and upregulation of 39 of 51 RNA degradation pathway genes in 7.6 mo testes compared to 12.8 mo testes (Figure 6, D1, D2). We performed RSEM on 1.9–3.6, 7.6 and 12.8 mo testes RNA-seq data sets and calculated the FPKM sum of all known mm10 genes (Figure 7D). We found that 7.6 mo testes contained a significantly lower amount of total mRNA, compared to 12.8 mo testes (Figure 7D, *t*-test, *P* = 0.01). This is despite the fact that we also observed upregulation of the basal transcription factors pathway in 7.6 mo testes with 25 of 33 basal transcription factors pathway genes upregulated in 7.6 mo testes compared to 1.9–3.6 mo testes and 30 of 33 basal transcription factors pathway genes upregulated in 7.6 mo testes compared to 12.8 mo testes (Figure 6, A2 and B2 and Supplementary Figure S10). These findings suggest that a shift in the balance between transcription and RNA biogenesis in the testis of 7.6 mo mice may be responsible for the observed upregulation in the number of expressed L1 loci in this age group without a corresponding increase in the total L1 mRNA (Figure 4A), explaining why the number of expressed L1 loci and overall L1 FPKM levels can function as independent variables.

### L1 mRNA expression is organ-specific in rats

To determine whether our observation of organ-specific L1 mRNA expression in mice is unique to the *mus musculus* species, we analyzed L1 mRNA expression in cytoplasmic RNA extracted from male rat organs. To account for the reported difference in the quality of genome assembly between the two species (mm10 genome assembly coverage is 20×, considerably higher than the 6× coverage of the rn6 assembly), we compared the ‘mappability’ of L1 sequences in rn6 and mm10 genomes. This allows us to measure the likelihood of finding unique alignments for RNA-seq reads originating from L1 mRNA transcripts in mice and rats. The ‘mappability’ of L1 sequences in the rn6 and mm10 genome assemblies were calculated by aligning *rattus norvegicus* and *mus musculus* paired-end DNA sequencing reads to the rn6 or mm10 genome using the same bowtie alignment parameters utilized in our RNA analysis pipeline (described in the Bioinformatic Analysis section of Materials and Methods). We then plotted the number of uniquely mapped DNA reads to each L1 locus in the mm10 and rn6 genome assemblies (Supplementary Figure S11A). We observe that the rn6 genome has a far steeper drop off in the number of DNA-seq reads able to uniquely align to each L1 locus compared to mm10, indicating that L1 loci are less ‘mappable’ in the rn6 genome.

**Figure 8.**
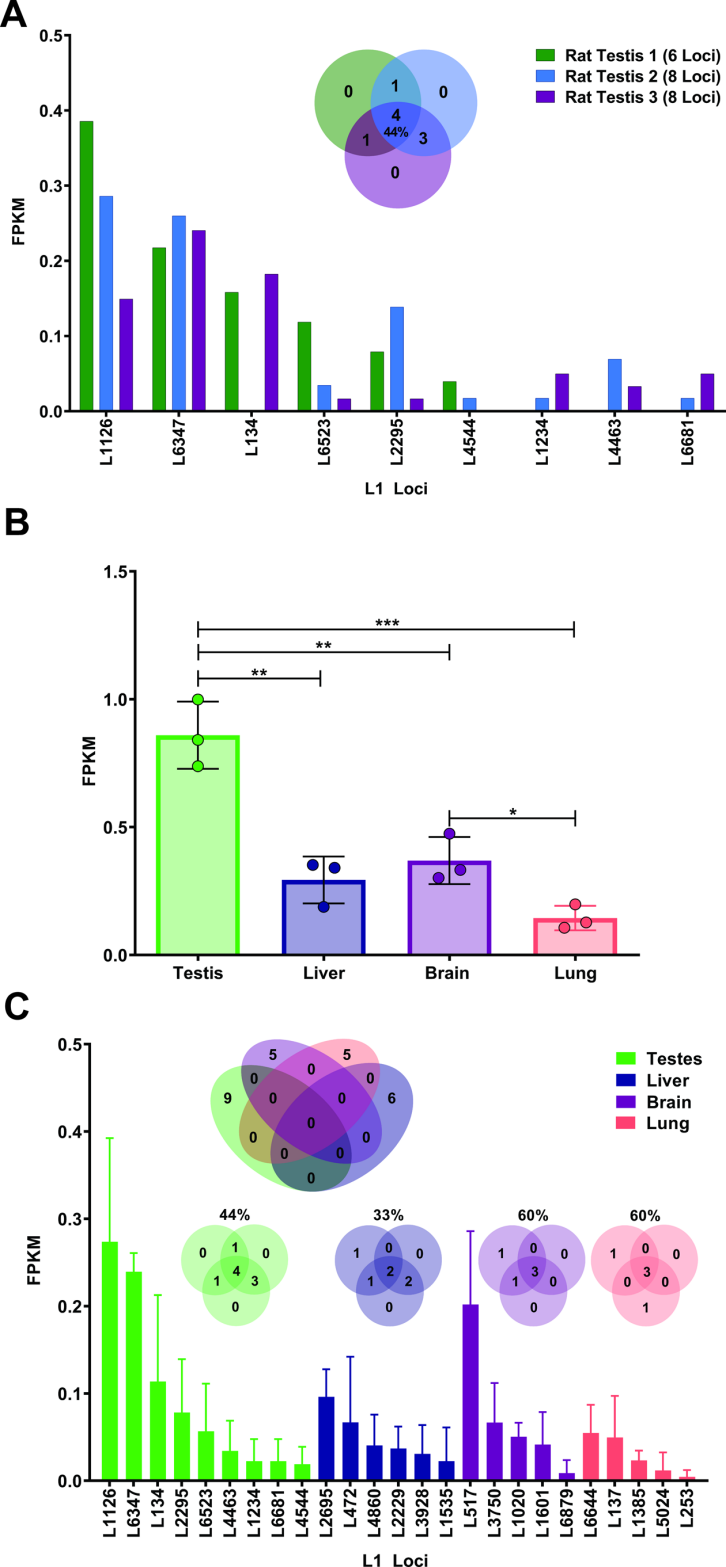
L1 mRNA expression in rat organs. (**A**) The bar graph shows expression levels (FPKM) of individual L1 loci expressed in testis collected from 4.8 mo male rats. The Venn diagram shows the number of loci shared between the three testis samples. The percent overlap is indicated in the center of the diagram. (**B**) A bar graph represents mean levels of total L1 expression with error bars showing standard deviation in rat testis, livers, brains and lungs. (**C**) A bar graph of all expressed L1 loci identified in different rat organs. The bars represent the average expression level from 3 samples with error bars indicating standard deviation measured by FPKM. The Venn diagram shows the overlap between expressed L1 loci from the different organs.

Taking the reduced level of L1 ‘mappability’ in the rn6 genome into consideration, we next performed our RNA-Seq analysis in testes, brains, lungs, and livers collected from three 4.8 mo rats to quantify levels of L1 mRNA expression (36). This analysis determined that, similar to mice, rat testes have a significantly higher level of total L1 mRNA expression than livers (*t*-test, *P* = 0.004), brains (*t*-test, *P* = 0.006), or lungs (*t*-test, *P* = 0.0009) (Figure 8A and B, Supplementary Figure S4 H-J). This analysis also shows that, similar to patterns of L1 loci expression observed in mouse organs, there was a high degree of overlap in L1 loci expressed in the same organ collected from different rats (44%, 33%, 60% and 60% in testis, liver, brain and lung, respectively; Figure 8A, Supplementary Figure S4H–J). L1 loci expression in organs collected from male rats shows strict organ-specificity, similar to our observations in male mice (Figures 2A and 8C).

We also observe that, due to the limited ‘mappability’ of the rn6 genome, fewer expressed L1 loci were identified in rats than the number of expressed L1 loci identified in mice (25 and 567, respectively). This difference made us wonder how variability in L1 locus mappability may influence the ability to align L1 reads to unmappable regions and measure L1 FPKM levels between samples. To address this question, we corrected our RNA-seq FPKM values for differences in mappability to observe whether mappability differences between loci would alter our conclusions. We mappability-corrected FPKM values from our male mouse RNA-seq analysis by scaling the number of aligned reads for each locus to 400 reads, the average number of reads for a fully ‘mappable’ L1 locus, and multiplying the number by the original FPKM value (Supplementary Figure S11A). This equation is described in more detail in the *Calculating mappability correction* section of the Materials and Methods. Normalizing to the ‘mappability’ of each locus did not change our comparison of L1 expression between organs and the mappability-corrected FPKM values for mouse testes remained significantly higher than mouse male livers, male brains, male lungs (*t*-test, *P* = 0.003, 0.003, 0.02, respectively, Supplementary Figure S11B).

## DISCUSSION

Detection of full-length L1 mRNA from individual loci is critical to understanding their impact on the host including potential to damage genomic DNA. To detect and measure L1 mRNA, methods used must be able to distinguish not only which locus is expressed, but whether that expression arises from the L1 promoter and is not a passive product of read-through transcription (33,34,39,41). Methods such as RT-PCR, northern blots, and many NGS-based approaches, fail to satisfy some or all of these criteria (91–94), leaving many important questions regarding L1 mRNA expression *in vivo* unresolved (4,34,42). Even methods that automate removal of passive incorporation of L1 sequences into cellular transcripts have difficulty reducing this background sufficiently to work on cells with low levels of L1 mRNA expression, as is typical for normal somatic cells (41,95–98). Additionally, analyses of either whole-cell RNA and/or non-stranded NGS preparations also decrease the sensitivity of L1 mRNA detection from specific loci (34,41,94). In this study, we have adapted our published method for the detection of rodent L1 mRNA expression utilizing unique mapping and visual validation of each potentially expressed L1 locus (34,36).

Patterns of L1 mRNA expression at the single-locus resolution are remarkably consistent within an organ, with testes from different mice sharing 85% of expressed L1 loci, male livers sharing 71%, male brains sharing 65%, male lungs sharing 52%, ovaries sharing 15%, uteri sharing 10%, female brains sharing 21%, and female lungs sharing 36%. (Figures 1 and 2, Supplementary Figures S4 and S6). Our *in vivo* analysis of L1 mRNA expression in multiple organs from 18 mice also found that the number of L1 loci differs greatly between organs, with mouse testes expressing significantly more L1 loci (i.e. 409 L1 loci expressed in testes versus 7 L1 loci expressed in liver, Figures 1, 2A, 3A). Similarly, 11 L1 loci were expressed in testis at a higher level than any other L1 locus in the other organs and overall L1 expression in testis is significantly higher than the other organs analyzed (Figures 1, 2, 3A and B). Despite a high degree of similarity within the same organ types, expressed L1 loci are rarely shared between different organ types collected from either male or female mice or rats (Figure 2). Analysis of organs dissected from male rats revealed similar patterns of L1 mRNA expression including a high degree of organ-specificity and enrichment of L1 expression in rat testes (Figure 8). This similarity in relative L1 mRNA expression patterns between mice and rat organs is consistent with findings in human cell lines (33,34), supporting that expression of individual L1 loci is controlled by their local transcriptional environment. One example would be the epigenetic reprogramming to form induced pluripotent stem cells (iPSCs) that leads to very high levels of L1 expression in reprogrammed cells relative to the parental cells (99). Similar findings were also shown for LTR retroelements (100) suggesting common principles across mobile element families.

If the sequence or epigenetic architecture of genomic domains is responsible for L1 regulation, we would expect that expressed L1 loci would be enriched in the vicinity of expressed genes that might result in the overall domain being more active. The fraction of expressed L1 loci found in genes is quite variable among organs (10–63%, Figure 5A). We observed that 28% of expressed L1 loci in testis occurred within a gene (Figure 5A) and of the genes containing an expressed L1 locus in testis, all, but one gene, are expressed (Figure 5B). Further analysis determined that expression levels of these L1 loci did not correlate with the levels of expression of the respective gene (Pearson correlation, *r* = 0.1030, Figure 5B). Our findings suggest that although the open chromatin state of a genomic region may catalyze expression of select L1 loci, it is not the only feature that defines their expression, or level of expression. It is likely that those expressed L1 elements that are present in genomic regions outside of expressed genes are also in more favorable chromatin domains compared to L1 loci that are not expressed. Our findings raise questions of why many other L1 loci that are present in expressed genes remain transcriptionally inert and why the proportion of expressed L1 loci present in genes varies significantly between organs (Figure 5). So far, all of our findings regarding L1 mRNA expression patterns *in vivo* and in cell lines (4,33–35) are most consistent with mobile elements being influenced by the cell-specific epigenetic patterns in the regions of the genome in which they find themselves.

Although similarities in L1 mRNA expression between organs of the same type are not surprising based on previous findings (4,33,34), it is worth considering the multiple cell types that make up each organ and how the different cell types are coordinated to produce consistent patterns of L1 mRNA expression at the organ level. For instance, either the most abundant cell type or the cell type with the highest level of L1 expression are likely to dominate the observed pattern. Developing appropriate custom tools for analysis of L1 mRNA expression at a single-locus resolution in individual cells will be the next and final level of an in-depth understanding of L1 transcriptome diversity and its contribution to somatic and germline retrotransposition in individual cells. Such studies would be of interest because it has been reported that only a subset of human neurons harbors a *de novo* somatic integration event (101,102). However, it is not known whether this rate of somatic mobilization results from permissive L1 expression in a few neurons or whether all cells express L1 mRNA and the rare integration events are an indicator of efficient suppression at the integration steps of the L1 replication cycle.

The ability to monitor changes in both the levels of L1 mRNA expression and numbers of expressed L1 loci allowed us to discover unique and dynamic variations that occur with age in different organs. We found that L1 mRNA expression and the number of L1 loci expressed can change independently with age (Figure 4, Supplementary Figure S6). Testes and male livers exhibited significant increases in the number of expressed L1 loci at both 7.6-months-old and 22.3-months-old (Figure 4, Supplementary Figure S6). While we do not yet understand the mechanism(s) underlying this complicated behavior, we have identified several key pathways that are upregulated in the testes of the 7.6 months-old mice that may be relevant. We see upregulation of basal transcription factor pathways (Figure 6 and Supplementary Figure S10) that might help more L1 loci express. In addition, upregulation of the RNA degradation pathway could explain why higher numbers of expressed loci do not lead to higher overall expression (Figures 6 and 7). Our data suggest that changes in patterns of L1 mRNA expression with age is complex and is a sum of genomic transcriptional environment changes influencing L1 loci, as well as pathway changes in various cell types that may synergize or antagonize one another relative to L1 locus expression. Our findings contrast with previous RT-PCR-based studies (92,93), which did not discriminate between L1 mRNA and passively transcribed L1 segments and did not provide locus-specific resolution (33,34). As a result, their findings are consistent with our analysis of background L1 sequences that are masking the signal from authentic L1 mRNA (Figure 3B, Supplementary Figure S5). The hypothesis that L1 mRNA expression increases with age has been discussed for decades (3,4,93,103–106). However, technical limitations associated with detection of repetitive sequences and low levels of endogenous L1 mRNA expression hindered progress in addressing it experimentally. Therefore, our findings provide the first rigorous experimental evidence that age-related changes in L1 mRNA expression are not uniform, rather L1 expression levels and profiles change with age in an organ- and sex-specific manner (Figure 4, Supplementary Figures S6 and S7). Our findings set the stage for future studies to understand whether the observed patterns of L1 expression are a cause or an effect of age-related changes within an organ or organism.

In addition to differences between organs and ages, we found that the levels and patterns of expressed L1 loci exhibited sex-specific differences. Male and female mice shared significantly more expressed L1 loci with their respective sex, compared to the L1 loci shared with the opposite sex (Supplementary Table S3). The number of expressed L1 loci shared between sexes changed with age, with the youngest age groups sharing the fewest number between sexes and significantly more expressed L1 loci shared between the 7.6–8.7-months and 12.8–16.5-months age groups (Supplementary Table S3). Even though the number of L1 loci shared between sexes increase with age from 2 L1 loci shared at 1.9–3.6-months to 21 L1 loci shared at 12.8–16.5-months, overall the two sexes share no more than 7% of expressed L1 loci across different age groups (Supplementary Table S3). We also compared L1 mRNA expression in equivalent organs, lungs and brains, between the two sexes. By comparing male lungs and brains with female lungs and brains within two different age groups, we found that male and female lungs shared 8–14% of expressed L1 loci and male and female brains shared 19–21% of expressed L1 loci (Figure 3C, Supplementary Table S3). Organ-specific comparative analysis revealed that significantly fewer L1 loci are shared between equivalent male and female organs, compared to the number of L1 loci shared amongst only female lungs or only male organs (Supplementary Table S3). In biological terms of L1 loci expression this means that lungs and brains collected from males or females are generally more similar to one another than to lungs and brains collected from the opposite sex.

Focusing only on reproductive organs, male testes share 85% of expressed L1 loci while ovaries and uteri share only 15% and 10% of expressed L1 loci, respectively (Figure 2). We considered that a significant difference in the number and levels of expressed L1 loci between these organs (409 loci in testis, 34 loci in ovary, 28 loci in uterus) could be contributing to this discrepancy (Figure 2 and Supplementary Table S3). However, this is likely not the case because male livers, brains, and lungs also express a low number and levels of L1 loci, yet they share 71%, 65%, and 52% of expressed L1 loci, respectively (Figure 2A). We consider that the variability within female organs could potentially be due to asynchronicity of the estrus cycle between female mice. Quantification of genes differentially expressed during the estrus cycle revealed that the female mice used in this study are in different stages of the estrus cycle (Figure 2, Supplementary Figure S8). Although this finding alone is inconclusive due to the limited number of animals analyzed in this study, it is intriguing to consider that changes in the transcriptional landscape due to hormonal fluctuations during estrus cycle may influence L1 mRNA expression in female reproductive organs as well as expression of a different subset of L1 loci compared to male reproductive organs. High levels of similarity of expressed L1 loci between male organs is true for both mice and rats with rat testes sharing 44% of expressed L1 loci, livers sharing 33%, brains sharing 60% and lungs sharing 60% (Figures 2A and 8C). This likeness between mice and rats suggests that their L1s respond to similar regulatory mechanisms even though mouse and rat L1 elements evolved different promoter sequences and L1 loci are present in different locations in their respective host genomes (107,108). Further speculation on this topic leads to thoughts of (i) whether L1 mRNA expression is sensitive to hormones primarily expressed in females, such as estrogen, and not as sensitive to hormones primarily expressed in males, such as testosterone, (ii) whether estrogen or testosterone have a suppressive effects on L1 expression, and (iii) whether this effect is unique to the mouse L1. A previous study has suggested that genes involved in estrogen signaling are also part of the LINE-1 regulatory network (109). These differences in L1 mRNA expression between reproductive and non-reproductive organs in males and females strongly support that there are both genetic and environmental triggers altering L1 mRNA expression patterns. They underscore the need to understand the underlying mechanisms governing this sexual dimorphism in L1 mRNA expression and assessment of the potential for adverse impact of these sex-specific differences in L1 expression on normal physiological processes and risk of developing diseases.

Overall, we found that expression of individual L1 loci in rodents is strikingly organ specific, with a strong level of sex specificity, establishing the first atlas of endogenous L1 mRNA expression in male and female mice. In addition, there are distinct patterns of increased heterogeneity in expression at different ages in different organs. Our cross organ, sex, and age comparisons establish a baseline of endogenous L1 mRNA expression under normal conditions that can be utilized to understand the potential of these elements to create differential DNA damage in different tissues with age or environmental exposures. Our findings set the stage for identifying mechanisms that permit expression of some but not other L1 loci and mechanisms that govern sexual dimorphism in L1 mRNA expression. These studies also permit comparative exploration of L1 mRNA expression patterns in other L1-harboring organisms to understand the roots of its evolutionary conservation or diversity.

## DATA AVAILABILITY

The raw data files and analysis files for RNA-seq in this manuscript are available through GEO: GSE158831. The aligned RNA-seq files are available through the following UCSC genome browser links: mm10, http://genome.ucsc.edu/cgi-bin/hgTracks?db=mm10&lastVirtModeType=default&lastVirtModeExtraState=&virtModeType=default&virtMode=0&nonVirtPosition=&position=chr12%3A56694976%2D56714605&hgsid=1015816353_RetTob8ARYR2ZUoUuEvAidjg9sZH and rn6, http://genome.ucsc.edu/cgi-bin/hgTracks?db=rn6&lastVirtModeType=default&lastVirtModeExtraState=&virtModeType=default&virtMode=0&nonVirtPosition=&position=chr1%3A80608553%2D80639261&hgsid=1015816387_WqXYk74yEffaVVS0vEHi5DAjFxPO.

## Supplementary Material

gkab369_Supplemental_Files
