# Supplementary material for: Organ-, sex- and age-dependent patterns of endogenous L1 mRNA expression at a single locus resolution
Source: Nucleic Acids Res. 2021 May 22;49(10):5813–31. doi: 10.1093/nar/gkab369 (PMC8191783; doi:10.1093/nar/gkab369)
Supplement: gkab369_Supplemental_Files [file gkab369_supplemental_files.zip › Supplemental Figures and Legends.pdf]

Supplemental Figure 1

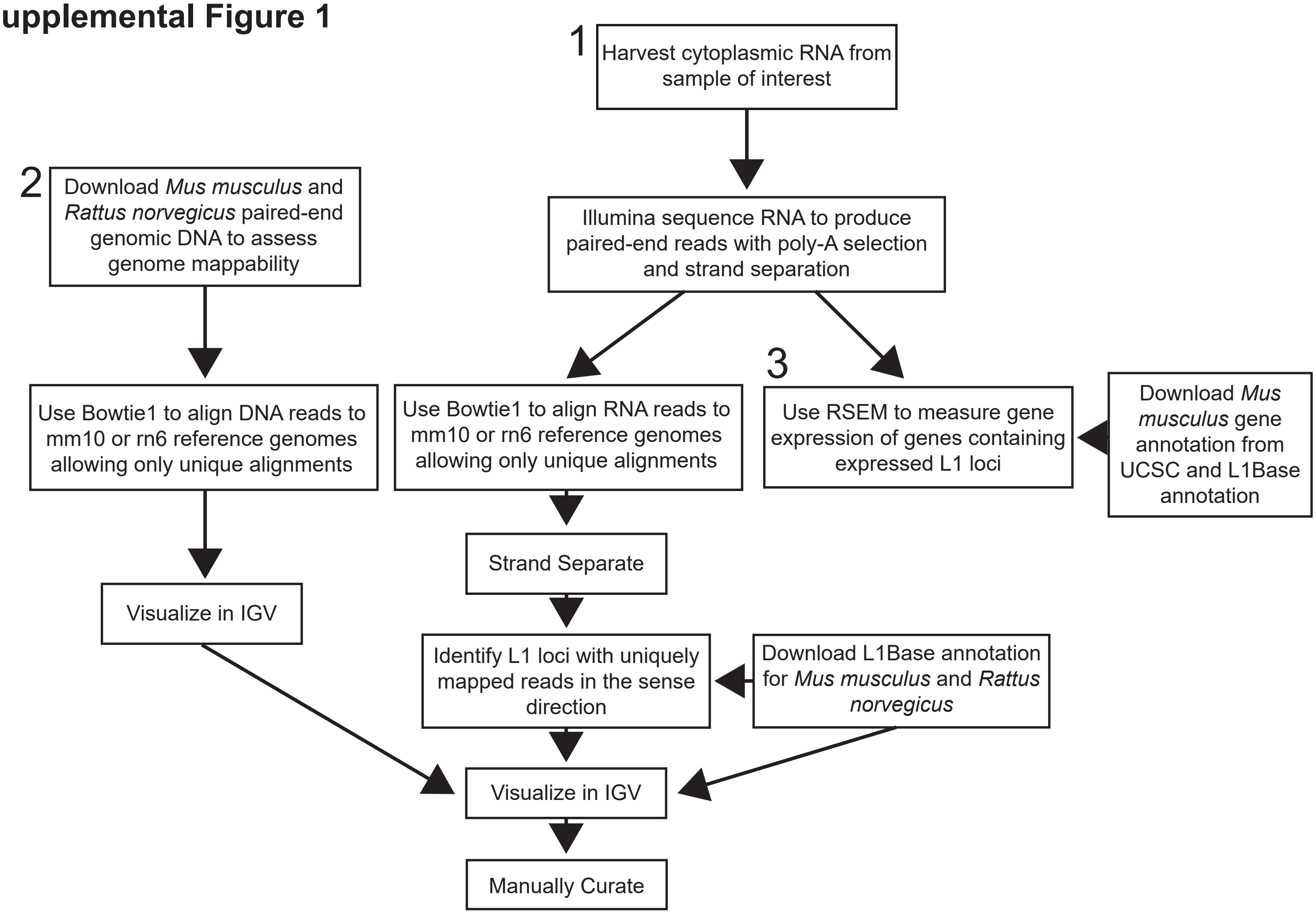

Supplemental Figure 1. Schematic describing steps for identifying L1 expression at the locus specific level. (1) Cytoplasmic RNA is harvested from the sample of interest and paired-end sequenced with poly-A selection and strand separation. Bowtie1 is used to align RNA-seq reads to the appropriate reference genome, allowing only unique alignments to be reported. Alignments are strand separated and visually validated as authentic L1 expression in IGV using a list of annotated L1 loci from L1Base and mappability of the appropriate genome derived from whole genome sequencing. (2) To obtain genome mappability, whole genome sequencing is downloaded for the appropriate species and aligned using Bowtie1 and the same parameters as used for the RNA-seq alignment. The mappability is then imported in IGV to be used while validating L1 expression. (3) RNA-seq reads may be analyzed using RSEM to determine expression levels of all genes. From the RSEM analysis, genes that contain L1 loci are extracted.

Supplemental Figure 2

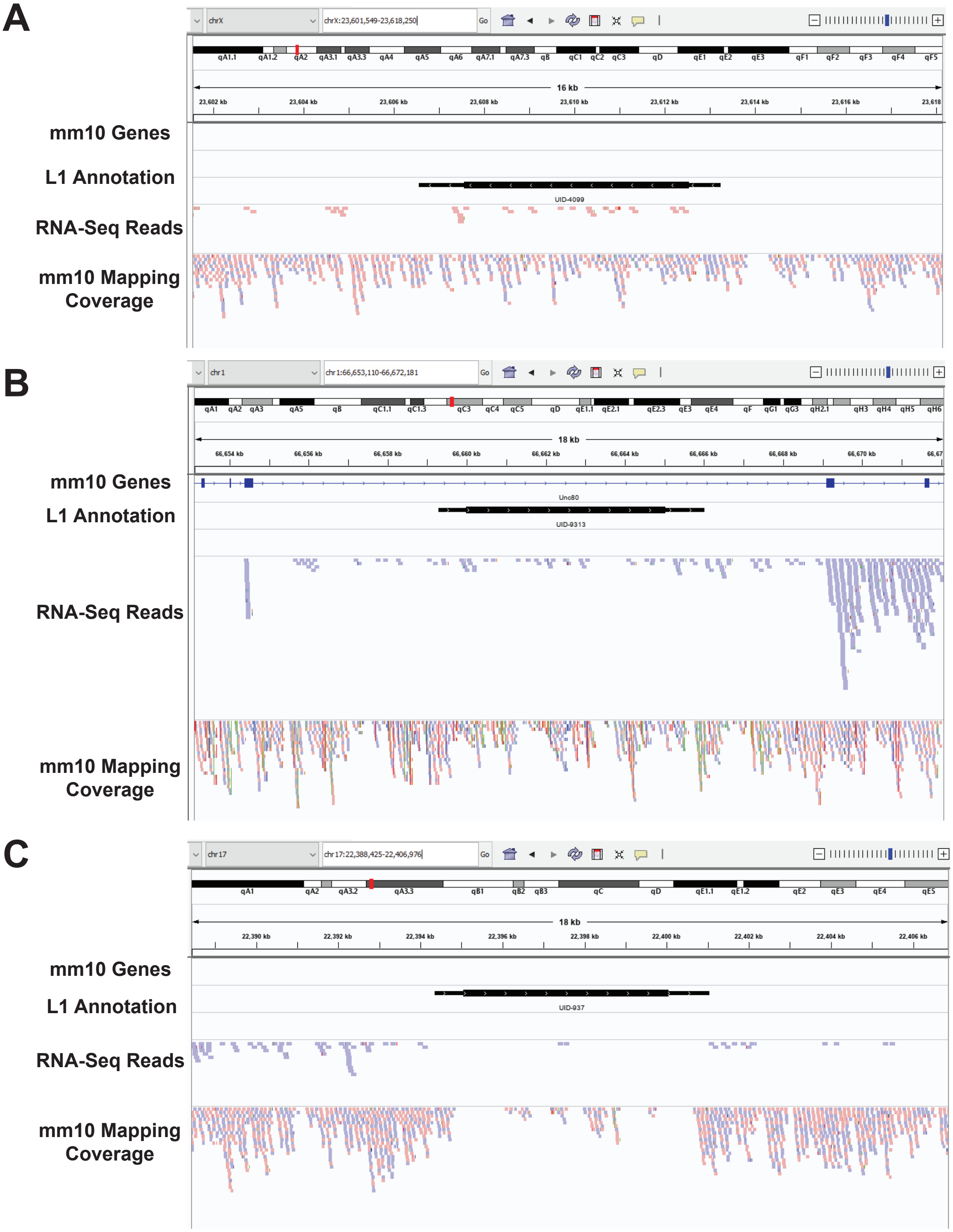

Supplemental Figure 2. Examples of manual curation of L1 loci in the Integrative Genomics Viewer (IGV). Top to bottom rows in each image: chromosome coordinates, chromosome with the position of the displayed region indicated by a red rectangle, mm10 genes, L1 annotation of full-length mm10 L1s from L1Base (thick line indicates open reading frame), paired-end RNA sequencing reads (blue for mRNA facing right and red for facing left), and mappability coverage of the mm10 genome. **(A)** An example of an authentically expressed L1 locus. UID-4099 is authentically expressed because it does not occur in a gene, does not have reads upstream of the L1 promoter, contains at least 10 reads in the same orientation as the L1 and spans a region of high mappabity in the mm10 genome. **(B)** An example of an L1 locus rejected by manual curation as authentically expressed. UID-9313 is not authentically expressed because it occurs within a gene of the same orientation and many reads occur upstream of the L1 promoter, suggesting the expression originates from the gene (Unc80) mRNA. **(C)** An example of an L1 locus rejected by manual curation as authentically expressed. UID-937 is also judged as not expressed from the L1 promoter because reads occur directly upstream of the L1 promoter and very few reads occur within the L1 body and do not correlate with the mappability of this genomic region.

Supplemental Figure 3

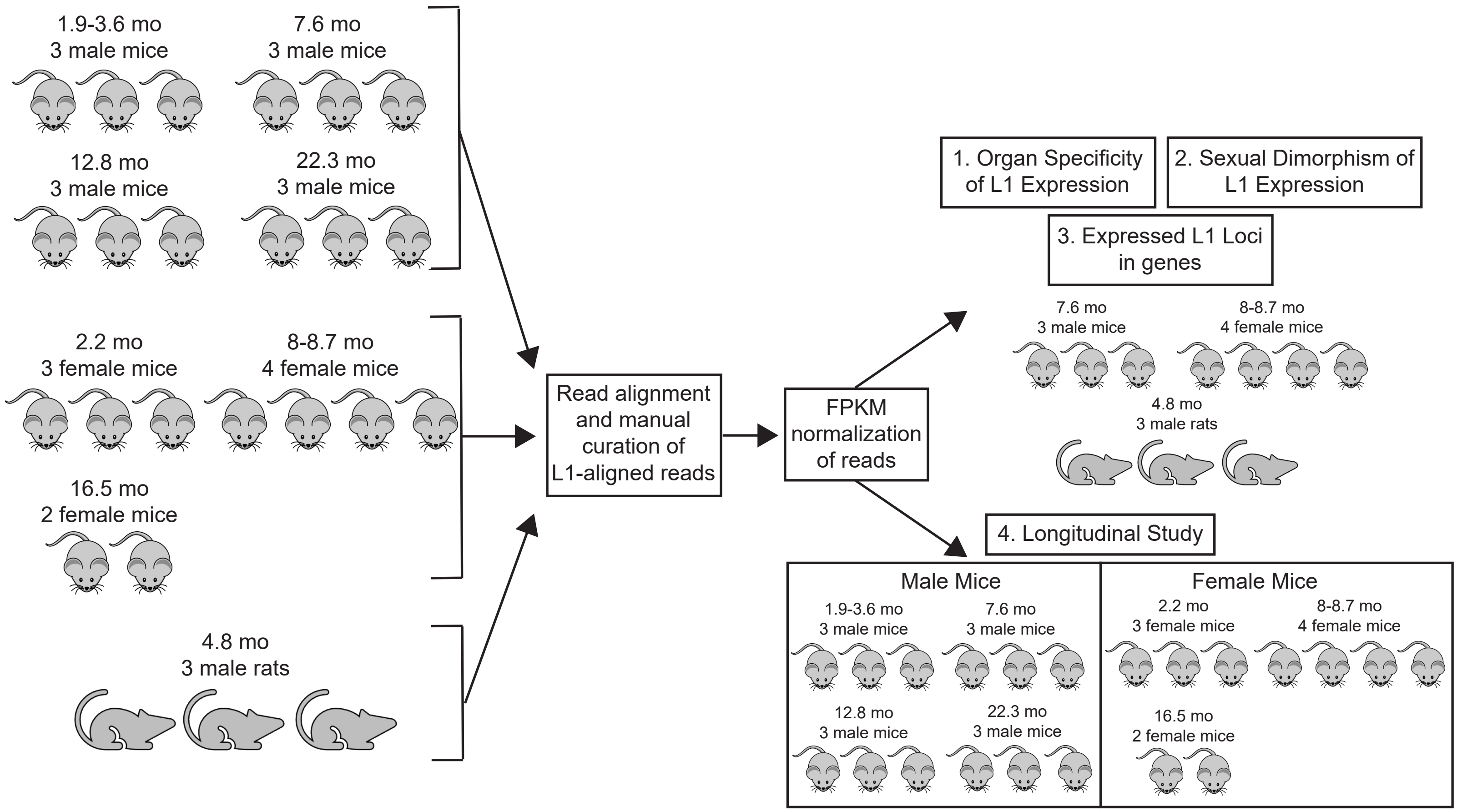

Supplemental Figure 3. Schematic of sample groups and the inclusion of groups in downstream analysis. Organs from 12 male mice, 9 female mice, and 3 male rats were used in this study. Cytoplasmic RNA was harvested from animal organs, sequenced, and aligned to either the mm10 or rn6 genome. L1 mRNA expression was validated using IGV. The numbers aligning to authentically expressed L1 loci were FPKM normalized. 7.6mo male mice, 8-8.7mo female mice, and 4.8mo male rats were used to determine the organ specificity of L1 expression. 7.6mo male mice and 8-8.7mo female mice were used to analyze sexual dimorphism of L1 expression and evaluate expression of L1 loci that occur within genes. Both male and female mice and all mouse age groups were used in the longitudinal study to establish age-specific patterns of L1 expression.

Supplemental Figure 4

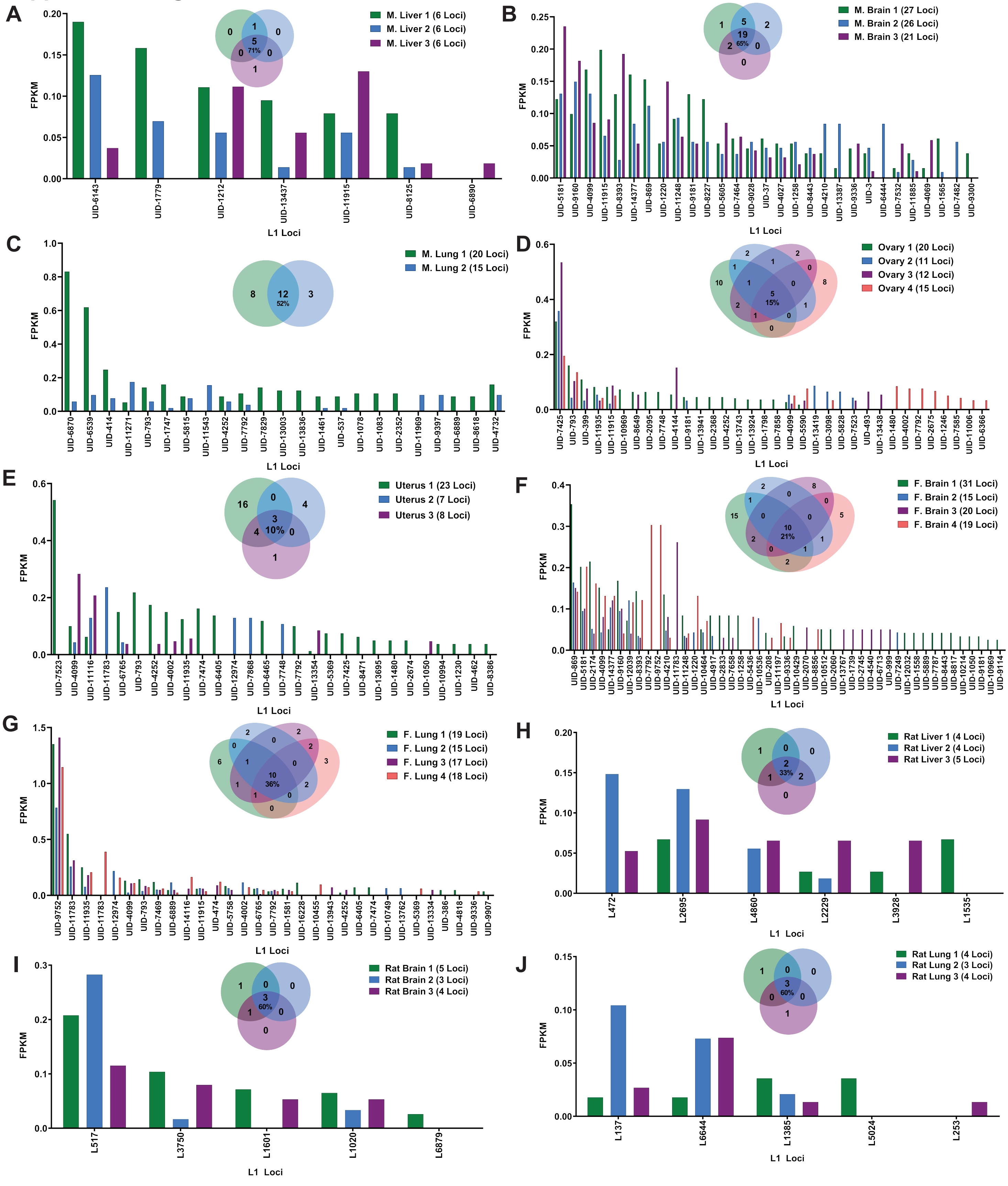

Supplemental Figure 4. Analysis of expressed L1 loci for male mouse samples, female mouse samples, and male rat samples. (A) A bar graph of mRNA expression levels from L1 loci identified to be expressed in livers dissected from three male mice 7.6 mo and a corresponding Venn diagram showing overlap between liver samples collected from these mice. **(B)** A bar graph of L1 mRNA expression levels from loci in three male mouse brains 7.6 mo and corresponding Venn diagram measuring overlap between 7.6 mo male brain samples. **(C)** A bar graph of L1 mRNA expression levels from loci in two male mouse lungs 7.6 mo and corresponding Venn diagram measuring overlap between 7.6 mo male lung samples. **(D)** A bar graph of L1 mRNA expression levels from loci in four mouse ovaries 8-8.6 mo and corresponding Venn diagram measuring overlap between 8-8.6 mo ovary samples. **(E)** A bar graph of L1 mRNA expression levels from loci in three mouse uteri 8-8.6 mo and corresponding Venn diagram measuring overlap between 8-8.6 mo uterus samples. **(F)** A bar graph of L1 mRNA expression levels from loci in four female mouse brains 8-8.6 mo and corresponding Venn diagram measuring overlap between 8-8.6 mo female brain samples. **(G)** A bar graph of L1 mRNA expression levels from loci in four female mouse lungs 8-8.6 mo and corresponding Venn diagram measuring overlap between 8-8.6-month female lung samples. **(H)** A bar graph of L1 mRNA expression levels from loci in three male rat livers 4.8 mo and corresponding Venn diagram measuring overlap between 4.8 mo rat liver samples. **(I)** A bar graph of L1 mRNA expression levels from loci in three male rat brains 4.8 mo and corresponding Venn diagram measuring overlap between 4.8 mo rat brain samples. **(J)** A bar graph of L1 mRNA expression levels from loci in male rat lungs 4.8 mo and corresponding Venn diagram measuring overlap between 4.8 mo rat lung samples.

Supplemental Figure 5

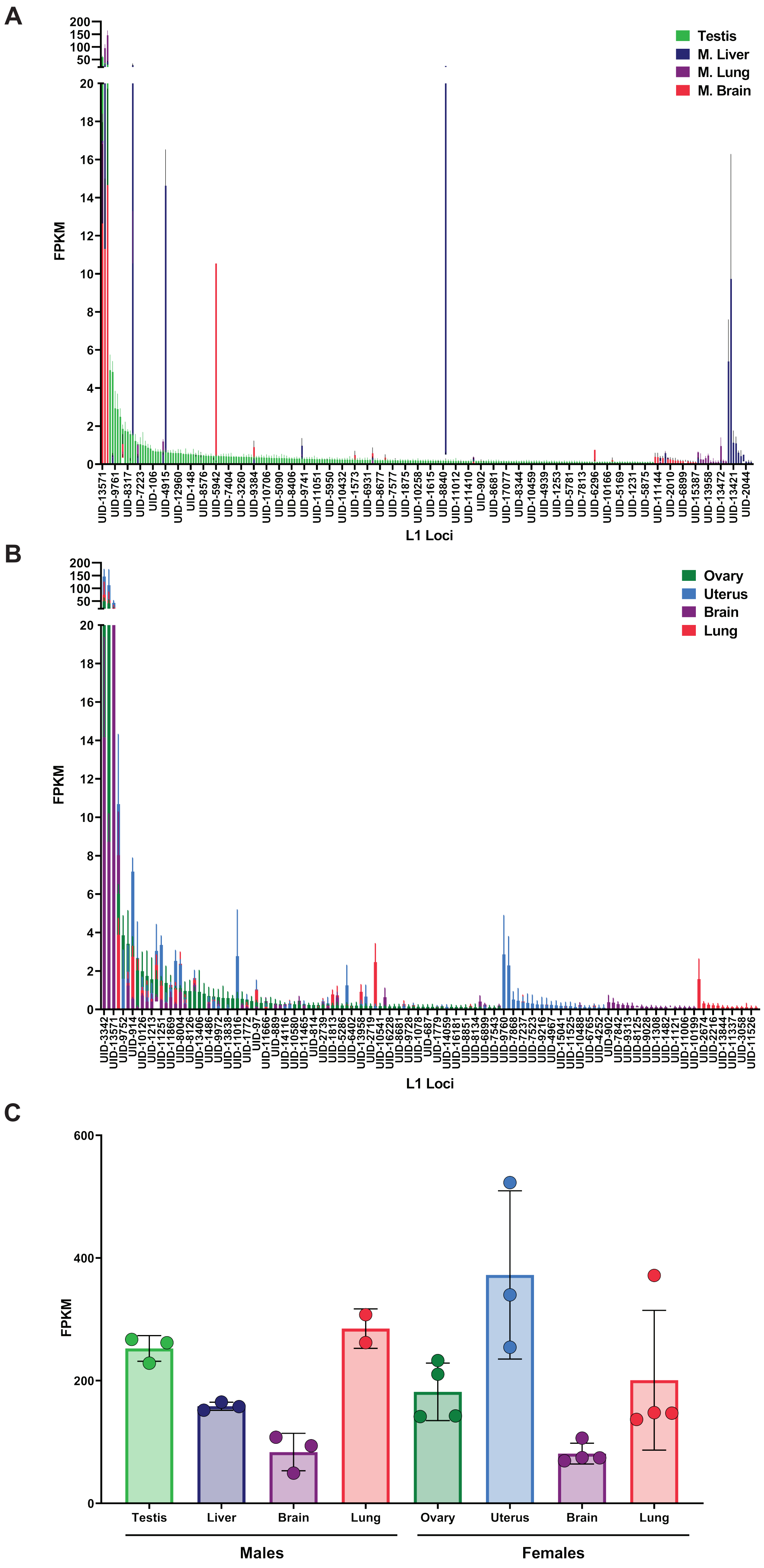

Supplemental Figure 5. Quantification of background L1 sequences detected in this study as reads mapping to L1 loci that are not authentically expressed, I.e. L1 sequences that are incorporated in cellular mRNA during cellular gene transcription. (A) Background L1 sequences present in transcription identified in male mouse organs. Sequence levels measured by FPKM. Error bars represent standard deviation produced by averaging L1 sequence levels across each set of samples. **(B)** Background L1 expression levels in female mouse organs. Background expression levels measured by FPKM. Bars represent standard deviation produced by averaging background expression levels across each set of organ samples. **(C)** Total background L1 expression levels for all male mouse organs and all female mouse organs with standard deviation error bars. Note that all samples had much higher background expression than the L1 mRNA expression in Figure 3B.

Supplemental Figure 6

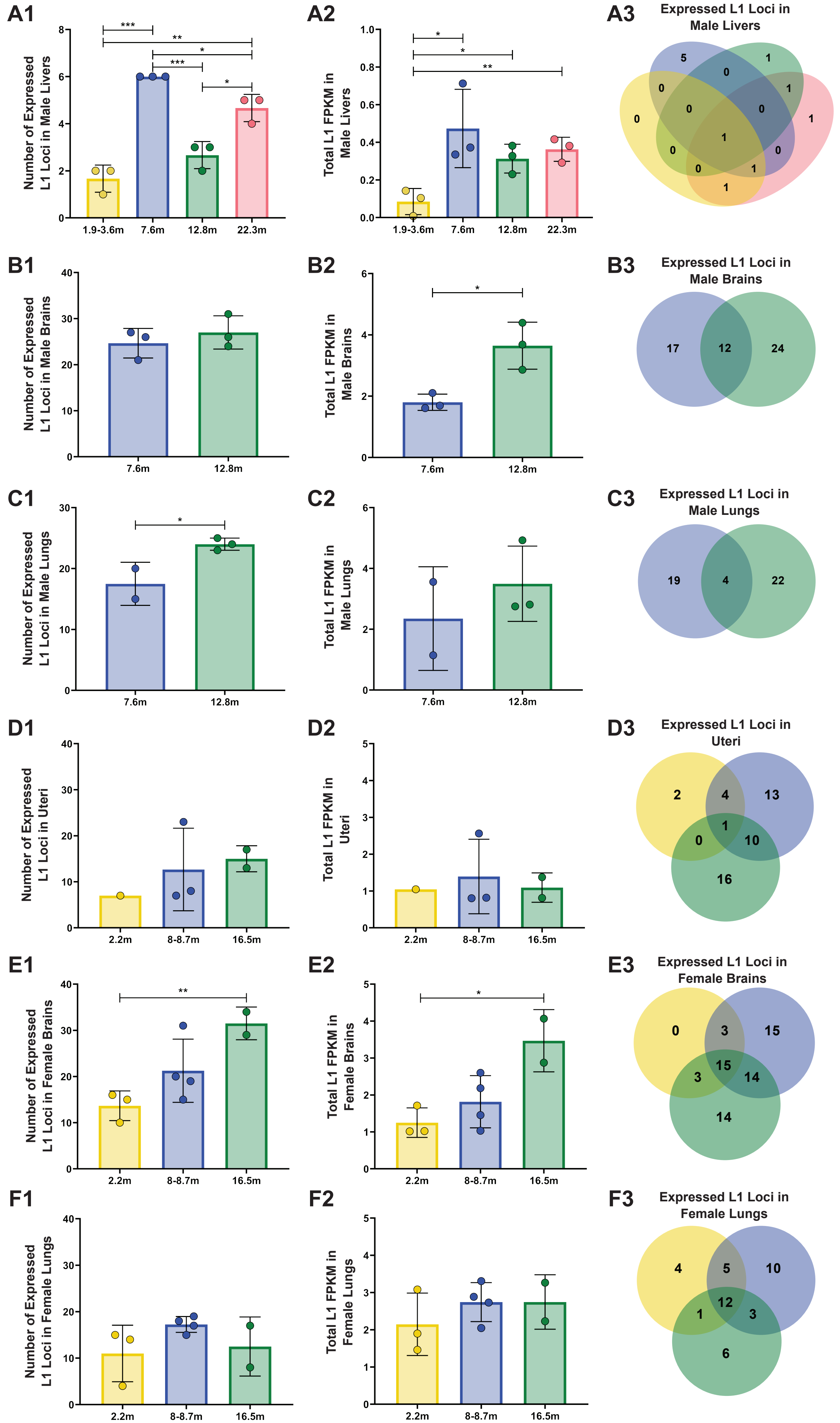

Supplemental Figure 6. Analysis of L1 mRNA expression across different ages for male and female mouse organs. (A) L1 mRNA expression in male livers. **(A1)** Average number of L1 loci expressed in livers collected from males at 1.9-3.6 mo, 7.6-months, 12.8 mo, and 22.3 mo (*t*-test, *** ≤ 0.005, ** ≤ 0.01, * ≤ 0.05). **(A2)** Total L1 mRNA expression at different ages for male livers. The sum of all expressed L1 loci were taken for samples across the indicated age groups in male livers (*t*-test, ** ≤ 0.01, * ≤ 0.05). **(A3)** A Venn diagram showing the overlap between expressed L1 loci from male livers of different ages. **(B)** L1 expression in male brains. **(B1)** Average number of L1 loci expressed at different ages for male brains. Numbers of expressed L1 loci were counted for male brains 7.6 mo and 12.8 mo. **(B2)** Total L1 expression at different ages for male brains. The sum of all expressed L1 loci were taken for samples across the indicated age groups in male brains (*t*-test, * ≤ 0.05). **(B3)** A Venn diagram showing the overlap between expressed L1 loci from male brains of different ages. **(C)** L1 expression in male lungs. **(C1)** Average number of L1 loci expressed at different ages for male lungs. Numbers of expressed L1 loci were counted for male lungs 7.6 mo and 12.8 mo (*t*-test, * ≤ 0.05). **(C2)** Total L1 expression at different ages for male lungs. The sum of all expressed L1 loci were taken for samples across the indicated age groups in male lungs. **(C3)** A Venn diagram showing the overlap between expressed L1 loci from male lungs of different ages. **(D)** L1 expression in uteri. **(D1)** Average number of L1 loci expressed at different ages for uteri. Numbers of expressed L1 loci were counted for uteri 2.2 mo, 8-8.7 mo, and 16.5 mo. **(D2)** Total L1 expression at different ages for uteri. The sum of all expressed L1 loci were taken for samples across the indicated age groups in uteri. **(D3)** A Venn diagram showing the overlap between expressed L1 loci from uteri of different ages. **(E)** L1 expression in female brains. **(E1)** Average number of L1 loci expressed at different ages for female brains. Numbers of expressed L1 loci were counted for female brains 2.2 mo, 8-8.7 mo, and 16.5 mo (*t*-test, ** ≤ 0.01). **(E2)** Total L1 expression at different ages for female brains. The sum of all expressed L1 loci were taken for samples across the indicated age groups in female brains (*t*-test, * ≤ 0.05). **(E3)** A Venn diagram showing the overlap between expressed L1 loci from female brains of different ages. **(F)** L1 expression in female lungs. **(F1)** Average number of L1 loci expressed at different ages for female lungs. Numbers of expressed L1 loci were counted for female lungs 2.2 mo, 8-8.7 mo, and 16.5 mo. **(F2)** Total L1 expression at different ages for female lungs. The sum of all expressed L1 loci were taken for samples across the indicated age groups in female lungs. **(F3)** A Venn diagram showing the overlap between expressed L1 loci from female lungs of different ages.

Supplemental Figure 7

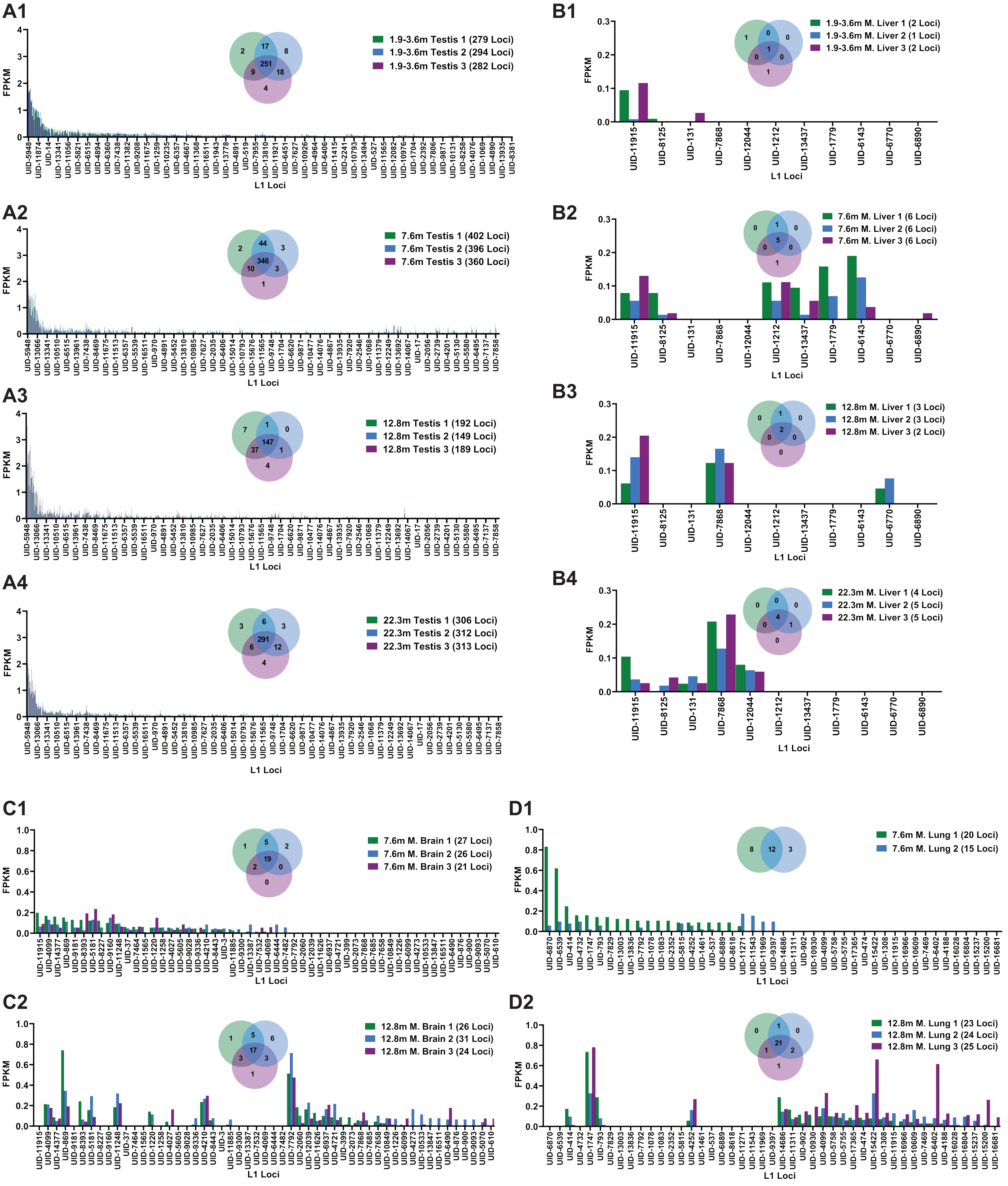

Supplemental Figure 7 (cont.)

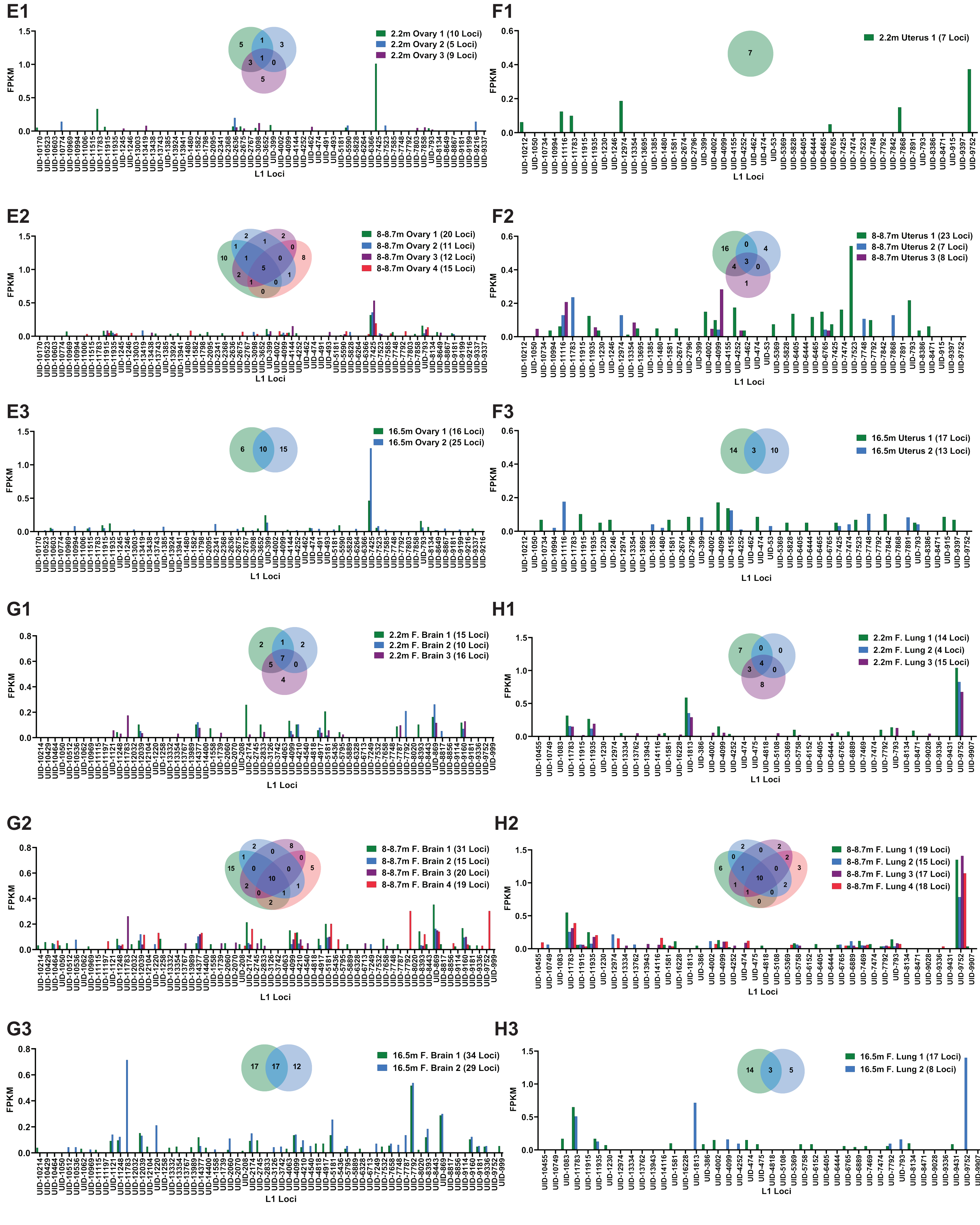

Supplemental Figure 7. Expression levels at specific L1 loci for each organ and age group. Each graph shows the FPKM level for each expressed L1 locus and a Venn diagram represents the number of shared expressed L1 loci between samples in each age group. **(A)** L1 mRNA locus-specific expression levels for testes collected from 1.9-3.6 mo mice **(A1)**, L1 mRNA locus-specific expression levels for testes collected from 7.6 mo mice **(A2)**, L1 mRNA locus-specific expression levels for testes collected from 12.8 mo mice **(A3)**, and L1 mRNA locus-specific expression levels for testes collected from 22.3 mo mice **(A4)**. **(B)** L1 mRNA locus-specific expression levels for livers collected from 1.9-3.6 mo male mice **(B1)**, L1 mRNA locus-specific expression levels for livers collected from 7.6 mo male mice **(B2)**, L1 mRNA locus-specific expression levels for livers collected from 12.8 mo male mice **(B3)**, and L1 mRNA locus-specific expression levels for livers collected from 22.3 mo male mice **(B4)**. **(C)** L1 mRNA locus-specific expression levels for brains collected from 7.6 mo male mice **(C1)** and L1 mRNA locus-specific expression levels for brains collected from 12.8 mo male mice **(C2)**. **(D)** L1 mRNA locus-specific expression levels for lungs collected from 7.6 mo male mice **(D1)** and L1 mRNA locus-specific expression levels for lungs collected from 12.8 mo male mice **(D2)**. **(E)** L1 mRNA locus-specific expression levels for ovaries collected from 2.2 mo mice **(E1)**, L1 mRNA locus-specific expression levels for ovaries collected from 8-8.7 mo mice **(E2)**, and L1 mRNA locus-specific expression levels for ovaries collected from 16.5 mo mice **(E3)**. **(F)** L1 mRNA locus-specific expression levels for uteri collected from 2.2 mo mice **(F1)**, L1 mRNA locus-specific expression levels for uteri collected from 8-8.7 mo mice **(F2)**, and L1 mRNA locus-specific expression levels for uteri collected from 16.5 mo mice **(F3)**. **(G)** L1 mRNA locus-specific expression levels for brains collected from 2.2 mo female mice **(G1)**, L1 mRNA locus-specific expression levels for brains collected from 8-8.7 mo female mice **(G2)**, and L1 mRNA locus-specific expression levels for brains collected from 16.5 mo female mice **(G3)**. **(H)** L1 mRNA locus-specific expression levels for lungs collected from 2.2 mo female mice **(H1)**, L1 mRNA locus-specific expression levels for lungs collected from 8-8.7 mo female mice **(H2)**, and L1 mRNA locus-specific expression levels for lungs collected from 16.5 mo female mice.

Supplemental Figure 8

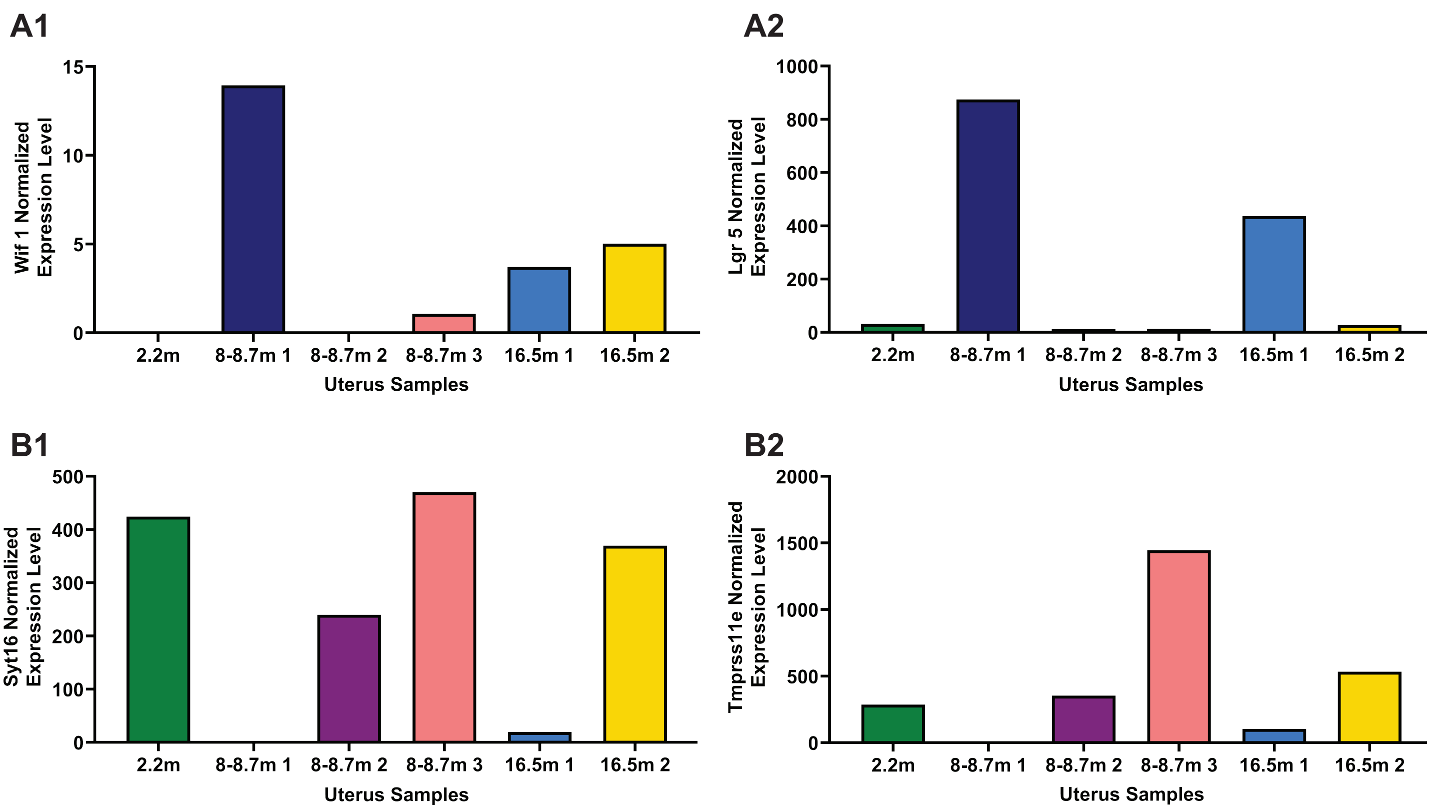

Supplemental Figure 8. Expression of estrus cycle regulated genes in uterus samples. (A-B) Expression levels of genes that fluctuate with the estrus/proestrus cycle in different uterus samples. **(A1)** Expression levels for Wif1, a gene expressed in the estrus phase. **(A2)** Expression levels for Lgr5, a gene expressed in the estrus phase. **(B1)** Expression levels for Syt16, a gene expressed in the proestrus phase. **(B2)** Expression levels for Tmprss11e, a gene expressed in the proestrus phase.

Supplemental Figure 9

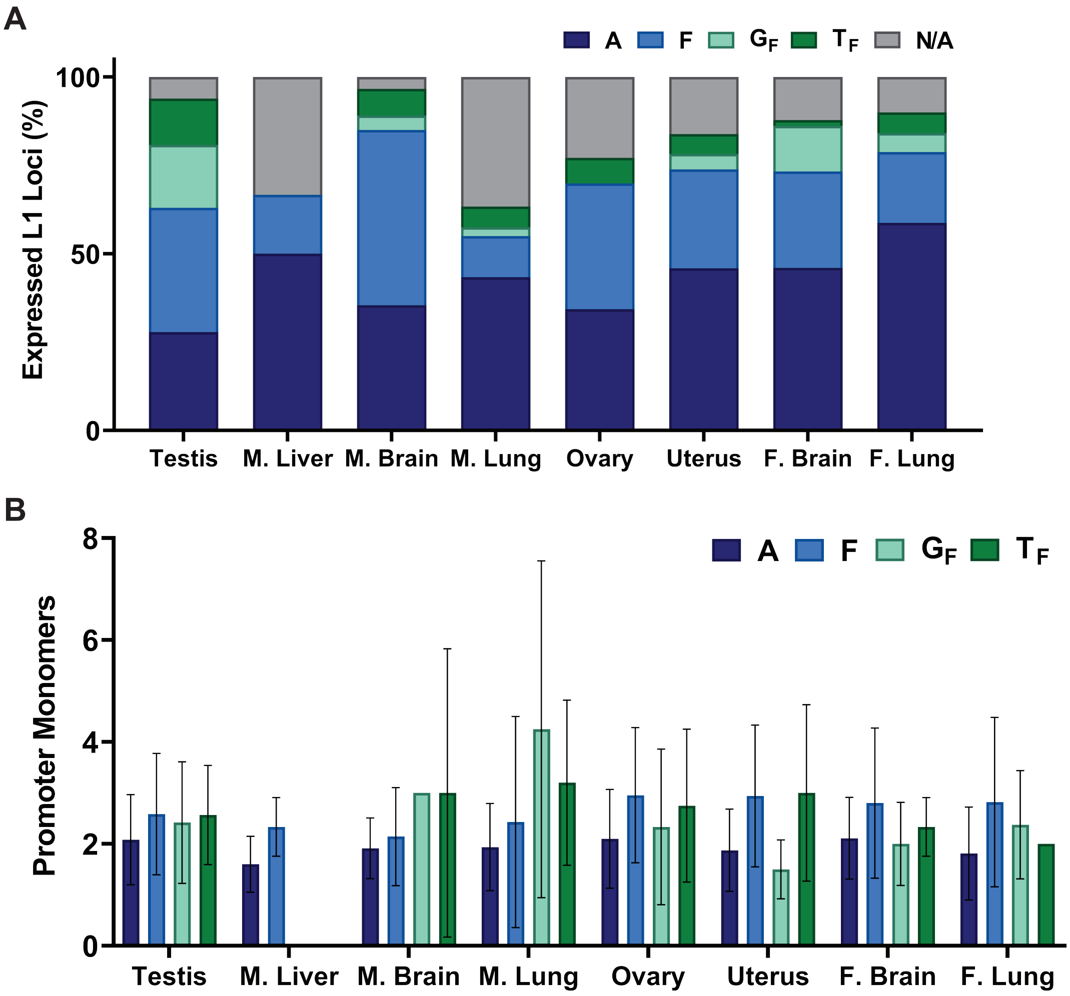

Supplemental Figure 9. Mouse L1 subfamily distribution of expressed L1 loci and number of monomers in expressed L1 loci promoters. (A) The percentage of L1 loci belonging to mouse L1 subfamilies (A, F, G_F, or T_F) for each organ type is shown. **(B)** The number of promoter monomers in each expressed L1 locus for each L1 subfamily (A, F, G_F, or T_F) is shown per organ. The error bars represent the standard deviation.

Supplemental Figure 10

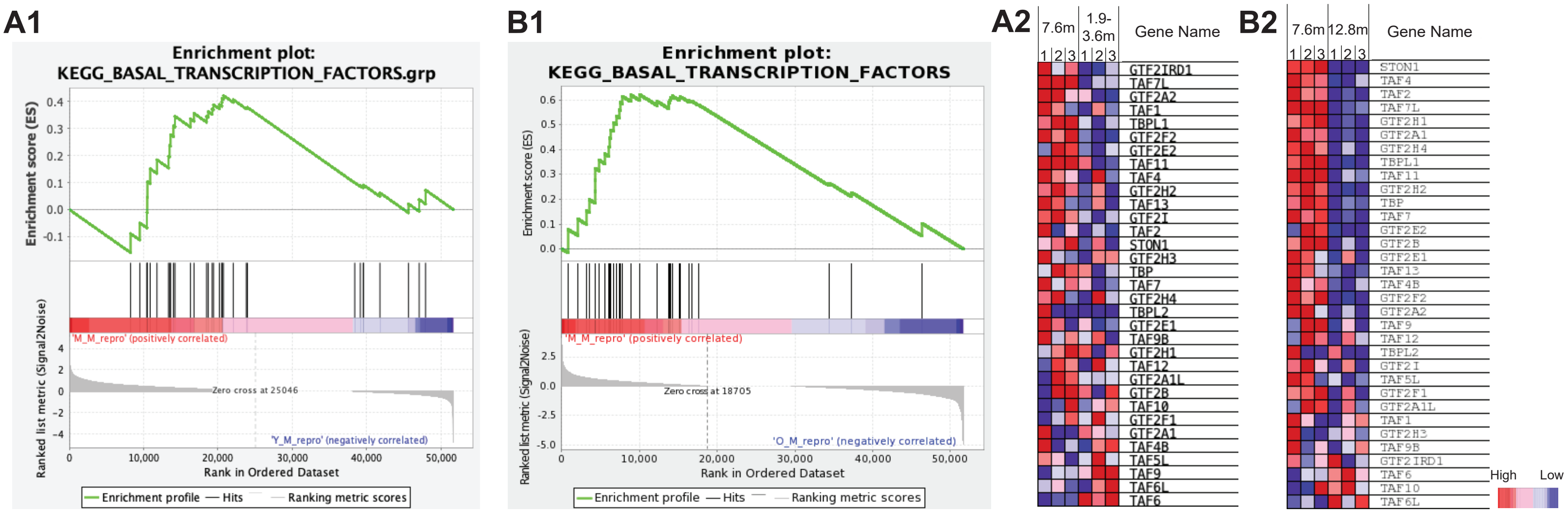

Supplemental Figure 10. Gene set enrichment analysis for basal transcription factors in 7.6 mo testes. (A1) Basal transcription factors gene set enrichment plot for 7.6 mo testes compared to 1.9-3.6 mo testes. **(A2)** Heatmaps for genes in the basal transcription factors pathway comparing expression between 7.6 mo and 1.9-3.6mo testes. **(B1)** Basal transcription factors gene set enrichment plot for 7.6 mo testes compared to 12.8 mo testes. **(B2)** Heatmaps for genes in the basal transcription factors pathway comparing expression between 7.6 mo and 12.8 mo testes.

Supplemental Figure 11

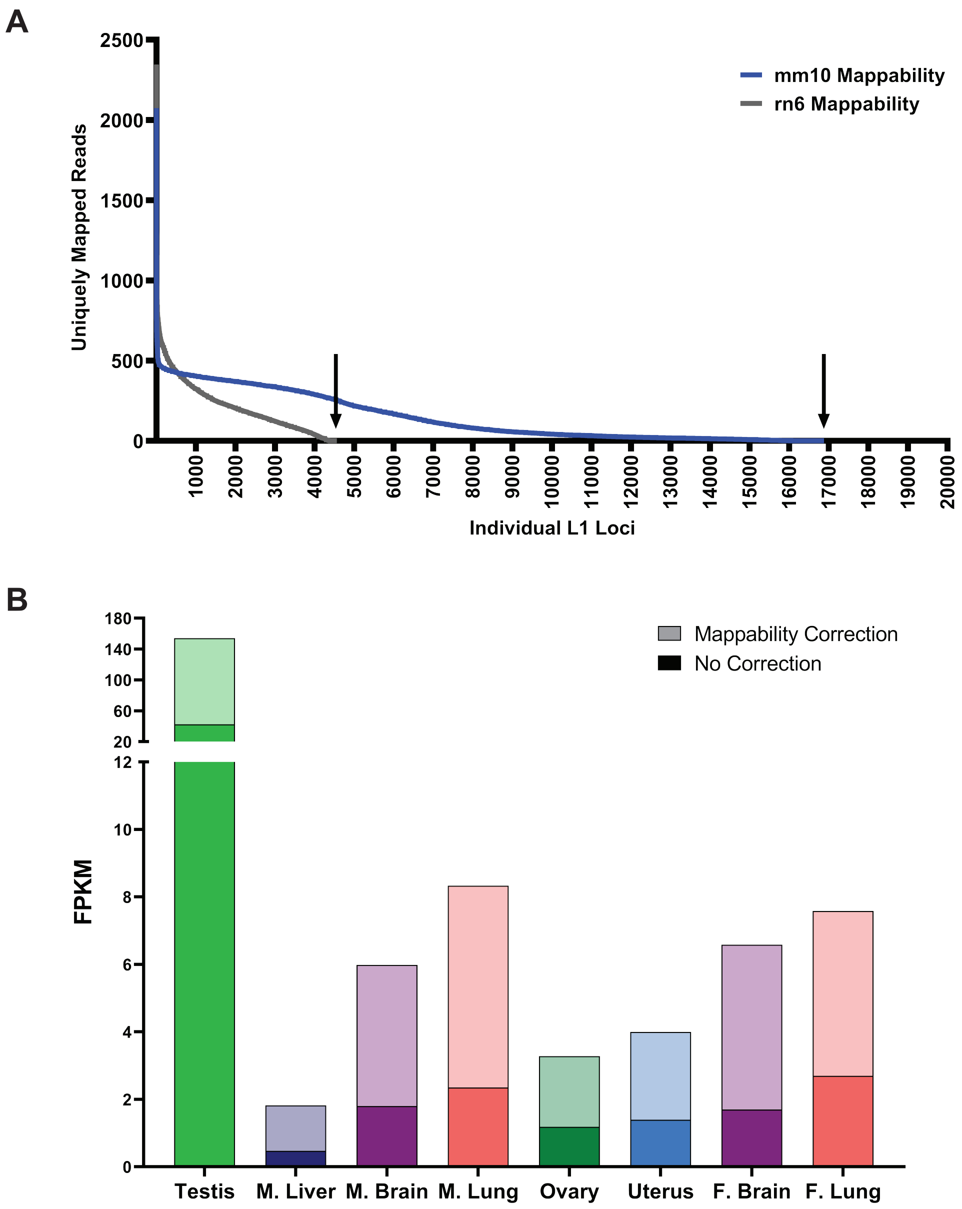

Supplemental Figure 11. Mappability considerations for L1 loci in rodent genomes. (A) Number of mapped reads from genomic DNA per annotated L1 locus in mm10 genomes and rn6 genomes. Arrows indicate where mapped reads drop-off for each genome assembly. **(B)** L1 mRNA expression level corrected for relative read mappability for each specific L1 locus identified to be expressed in different mouse organs. Corrected values are shown in light colors for comparison with uncorrected values shown in dark colors.
