# Supplementary material for: Organ-, sex- and age-dependent patterns of endogenous L1 mRNA expression at a single locus resolution
Source: Nucleic Acids Res. 2021 May 22;49(10):5813–31. doi: 10.1093/nar/gkab369 (PMC8191783; doi:10.1093/nar/gkab369)
Supplement: gkab369_Supplemental_Files [file gkab369_supplemental_files.zip › Supplemental File 3.docx]

mm10_RNAalign.sh script:

#!/bin/bash

#SBATCH --qos=normal

#SBATCH --job-name=mm10_RNASeq

#SBATCH -o mm10_RNASeq _OutputLog.txt

#SBATCH -e mm10_RNASeq _ErrorLog.txt

#SBATCH --time=12:00:00

#SBATCH --nodes=1 #nodes

#SBATCH --ntasks-per-node=12

#SBATCH --mem=128000

module load samtools/1.5

module load bedtools/2.27.1

prefix=${1%_[1-2].fq}

/path_to_bowtie-0.12.8/bowtie -p 10 -m 1 -S -y -v 3 -X 600 --chunkmbs 8184 /lustre/project/vperepe/apps/bowtieIndexes/mm10 -1 ${prefix}"_1.fq" -2 ${prefix}"_2.fq" | samtools view -hbuS - | samtools sort -o ${prefix}_bowtie_mm10_sorted.bam

samtools rmdup ${prefix}_bowtie_mm10_sorted.bam ${prefix}_rmdup_bowtie_mm10_sorted.bam

samtools view -h ${prefix}_rmdup_bowtie_mm10_sorted.bam | awk 'substr($0,1,1) == "@" || $2 == 83 || $2 == 163 {print}' | samtools view -bS - > ${prefix}_rmdup_bowtie_mm10_sorted_topstrand.bam

samtools view -h ${prefix}_rmdup_bowtie_mm10_sorted.bam | awk 'substr($0,1,1) == "@" || $2 == 99 || $2 == 147 {print}' | samtools view -bS - > ${prefix}_rmdup_bowtie_mm10_sorted_bottomstrand.bam

bedtools coverage -abam /lustre/project/vperepe/apps/mm10_orf2_nI_I_L1BASE_+700up-1000down_plus_numb.bed -b ${prefix}_rmdup_bowtie_mm10_sorted_topstrand.bam > ${prefix}_rmdup_bowtie_mm10_sorted_bowtie_tryhard_plus_top.txt

bedtools coverage -abam /lustre/project/vperepe/apps/mm10_orf2_nI_I_L1BASE_+700up-1000down_minus_numb.bed -b ${prefix}_rmdup_bowtie_mm10_sorted_bottomstrand.bam > ${prefix}_rmdup_bowtie_mm10_sorted_bowtie_tryhard_minus_bottom.txt

samtools index ${prefix}_rmdup_bowtie_mm10_sorted.bam

rn6_RNAalign.sh script:

#!/bin/bash

#SBATCH --qos=normal

#SBATCH --job-name=rn6_RNASeq

#SBATCH -o rn6_RNASeq_OutputLog.txt

#SBATCH -e rn6_RNASeq_ErrorLog.txt

#SBATCH --time=12:00:00

#SBATCH --nodes=1 #nodes

#SBATCH --ntasks-per-node=12

#SBATCH --mem=128000

module load samtools/1.5

module load bedtools/2.27.1

prefix=${1%_[1-2].fq}

/path_to_bowtie-0.12.8/bowtie -p 10 -m 1 -S -y -v 3 -X 600 --chunkmbs 8184 /lustre/project/vperepe/apps/bowtieIndexes/rn6 -1 ${prefix}"_1.fq" -2 ${prefix}"_2.fq" | samtools view -hbuS - | samtools sort -o ${prefix}_bowtie_rn6_sorted.bam

samtools rmdup ${prefix}_bowtie_rn6_sorted.bam ${prefix}_bowtie_rn6_sorted_rmdup.bam

samtools view -h ${prefix}_bowtie_rn6_sorted_rmdup.bam | awk 'substr($0,1,1) == "@" || $2 == 83 || $2 == 163 {print}' | samtools view -bS - > ${prefix}_bowtie_rn6_sorted_rmdup_topstrand.bam

samtools view -h ${prefix}_bowtie_rn6_sorted_rmdup.bam | awk 'substr($0,1,1) == "@" || $2 == 99 || $2 == 147 {print}' | samtools view -bS - > ${prefix}_bowtie_rn6_sorted_rmdup_bottomstrand.bam

bedtools coverage -a /lustre/project/vperepe/apps/R6_5UTRandL1BASEsorted_dedup_+.bed -b ${prefix}_bowtie_rn6_sorted_rmdup_topstrand.bam > ${prefix}_bowtie_rn6_sorted_tryhard_plus_top.txt

bedtools coverage -a /lustre/project/vperepe/apps/R6_5UTRLandL1BASEsorted_dedup_-.bed -b ${prefix}_bowtie_rn6_sorted_rmdup_bottomstrand.bam > ${prefix}_bowtie_rn6_sorted_tryhard_minus_bottom.txt

samtools index ${prefix}_bowtie_rn6_sorted_rmdup.bam
