# Supplementary material for: Organ-, sex- and age-dependent patterns of endogenous L1 mRNA expression at a single locus resolution
Source: Nucleic Acids Res. 2021 May 22;49(10):5813–31. doi: 10.1093/nar/gkab369 (PMC8191783; doi:10.1093/nar/gkab369)
Supplement: gkab369_Supplemental_Files [file gkab369_supplemental_files.zip › Supplemental Files captions.docx]

**Supplemental File 1. L1Base coordinates for all full length L1 loci in the mm10 genome.**

This file contains the coordinates, strand, and UID for all full length L1 loci in the mm10

genome.

**Supplemental File 2. L1Base coordinates for all full length L1 loci in the rn6 genome.** This file contains the coordinates, strand, and UID for all full length L1 loci in the rn6 genome.

**Supplemental File 3. Scripts for alignment and processing of RNA-sequencing reads to**

**mm10 and rn6 genomes.** This file contains computer cluster scripts for aligning RNAsequencing reads in a fastq file to either the mm10 or rn6 genome using the Bowtie aligner. These scripts also include downstream processing of alignment files using Samtools and BEDTools to extract stranded alignments and count the number of alignments that intersect with the list of annotated L1 loci.
