# Supplementary material for: Organ-, sex- and age-dependent patterns of endogenous L1 mRNA expression at a single locus resolution
Source: Nucleic Acids Res. 2021 May 22;49(10):5813–31. doi: 10.1093/nar/gkab369 (PMC8191783; doi:10.1093/nar/gkab369)
Supplement: gkab369_Supplemental_Files [file gkab369_supplemental_files.zip › Supplemental Table 1 legends.docx]

**Supplemental Table 1. A table containing the total number of L1 loci and reads mapping to L1 loci before and after manual curation.** This file contains tabs for an age key to match sample numbers with their age, manual curation numbers for all male samples, and manual curation numbers for all female samples used in this study.

**Supplemental Table 2. A table containing FPKM values for every expressed L1 mRNA locus reported in this study.** This file contains tabs for an Age Key to match sample numbers with their age and FPKM values and their corresponding L1 loci for male mouse organs, female mouse organs, and male rat organs.

**Supplemental Table 3. Expressed L1 loci shared between organs, sexes, and age groups.** This file contains a tab comparing expressed L1 loci between sexes by age and organ (Male v Female) and a tab comparing expressed L1 loci between age groups (Aging). Chi-Square analyses were performed to report significant differences in loci shared between different groups. Chi-Square tests and their corresponding dataset are color-coded.
